# Reciprocal F1 Hybrids of Two Inbred Mouse Strains Reveal Parent-of-Origin and Perinatal Diet Effects on Behavior and Expression

**DOI:** 10.1534/g3.118.200135

**Published:** 2018-08-31

**Authors:** Daniel Oreper, Sarah A. Schoenrock, Rachel McMullan, Robin Ervin, Joseph Farrington, Darla R. Miller, Fernando Pardo-Manuel de Villena, William Valdar, Lisa M. Tarantino

**Affiliations:** * Department of Genetics; † Bioinformatics and Computational Biology Curriculum, University of North Carolina, Chapel Hill, NC; ‡ Neuroscience Curriculum; § Genetics and Molecular Biology Curriculum; ** Lineberger Comprehensive Cancer Center, School of Medicine; †† Division of Pharmacotherapy and Experimental Therapeutics, Eshelman School of Pharmacy, University of North Carolina, Chapel Hill, NC

**Keywords:** C57BL/6J, NOD/ShiLtJ, nutritional deficiencies, imprinting, Bayesian mediation analysis

## Abstract

Parent-of-origin effects (POE) in mammals typically arise from maternal effects or imprinting. In some instances, such POE have been associated with psychiatric disorders, as well as with changes in a handful of animal behaviors. However, POE on complex traits such as behavior remain largely uncharacterized. Moreover, although both behavior and epigenetic effects are known to be modified by perinatal environmental exposures such as nutrient deficiency, the architecture of such environment-by-POE is mostly unexplored. To study POE and environment-by-POE, we employ a relatively neglected but especially powerful experimental system for POE-detection: reciprocal F1 hybrids (RF1s). We exposed female NOD/ShiLtJ×C57Bl/6J and C57Bl/6J×NOD/ShiLtJ mice, perinatally, to one of four different diets, then after weaning recorded a set of behaviors that model psychiatric disease. Whole-brain microarray expression data revealed an imprinting-enriched set of 15 genes subject to POE. The most-significant expression POE, on the non-imprinted gene *Carmil1* (a.k.a. *Lrrc16a*), was validated using qPCR in the same and in a new set of mice. Several behaviors, especially locomotor behaviors, also showed POE. Bayesian mediation analysis suggested *Carmil1* expression suppresses behavioral POE, and that the imprinted gene *Airn* suppresses POE on *Carmil1* expression. A suggestive diet-by-POE was observed on percent center time in the open field test, and a significant diet-by-POE was observed on one imprinted gene, *Mir341*, and on 16 non-imprinted genes. The relatively small, tractable set of POE and diet-by-POE detected on behavior and expression here motivates further studies examining such effects across RF1s on multiple genetic backgrounds.

It is well established that susceptibility to psychiatric disease arises from a combination of genetics and environment (Lee and Avramopoulos 2014). Less well-studied is the phenomenon that this susceptibility seems to vary depending on whether certain harmful alleles were carried by the mother or father (Davies *et al.* 2001; Isles and Wilkinson 2000), such that inherited susceptibility is subject to parent-of-origin effects (POE). Less studied still is the extent to which such POE depend upon environment during development, and therefore how much alternate environmental exposures could modulate a POE’s impact on psychiatric disease risk. A better understanding of POE and their environmental modifiers could lead to improved interpretation of existing studies, more effective experimental design, and could provide a basis for new public health interventions. Nonetheless, rigorous estimation of POE in humans is difficult, especially for complex traits such as behavior. Even in animal models it requires specialized experimental design attuned to POE biology.

## POE on psychiatric disease, behavior, and brain function

POE requires careful definition. The phrase “parent-of-origin effect” has sometimes been used interchangeably with “imprinting effects” [*e.g.* (Morison and Reeve 1998; Neugebauer *et al.* 2010)]. Here, however, akin to its use in Hager *et al.* (2008) and Lawson *et al.* (2013), we define POE as referring to *all genetically controlled* effects that vary depending on whether an inherited allele originated from the mother or father. Such POE can occur in mammals through multiple mechanisms, among them, maternal factors, paternal factors, and imprinting. These are briefly discussed below, along with previous findings relevant to behavior, brain development and function, and psychiatric disease.

### Maternal factors:

When maternal genetics controls a maternal factor that has no paternal equivalent (*e.g.*, gestational environment, maternal care, etc) and this in turn affects the offspring, then maternal genetic state is indirectly affecting offspring in a parent-of-origin dependent way. For example, embryonic transfer experiments in rodents have shown that uterine environment affects exploratory behavior (Francis *et al.* 2003); similarly, rodent cross-fostering experiments in rodents have shown that maternal care affects offspring traits such as hippocampal gene expression, oxytocin receptor binding, stress response, and exploratory behavior (Champagne *et al.* 2003; Weaver *et al.* 2006).

### Paternal factors:

POE might similarly arise though paternal factors (Braun and Champagne 2014). POE induced by paternal care is precluded by our experimental design, but POE can also arise in other ways: ablation of paternal accessory sex glands in golden hamsters has shown that seminal fluid levels affect offspring exploratory behavior (Wong *et al.* 2007); and injection of a *miR212/132* inhibitor into sperm cells has shown that sperm miRNA affect hippocampal long-term potentiation and (abeit weakly) cognitive ability (Benito *et al.* 2018).

### Imprinting:

Perhaps the best-characterized mechanism for POE (Lawson *et al.* 2013; Georges *et al.* 2003; Vrana *et al.* 2000; Wolf *et al.* 2014; Schultz *et al.* 2015), imprinting is an epigenetic process in which either the maternally or paternally inherited allele of certain genes is at least partially silenced (Crowley *et al.* 2015; Bartolomei and Ferguson-Smith 2011); as a result, reciprocal heterozygotes at imprinted loci differ in allelic expression ratio, and potentially in total expression. Imprinting can be developmental-stage (and tissue)-specific (Koerner *et al.* 2009; Prickett and Oakey 2012; Andergassen *et al.* 2017). Many imprinted genes are active (some exclusively) in the brain (Prickett and Oakey 2012), especially during embryogenesis (Wilkinson *et al.* 2007), and mouse knockout/mutation experiments have identified specific imprinted genes that affect brain size and organization (Wilkinson *et al.* 2007), control of nutritional resources (Bartolomei and Ferguson-Smith 2011), maternal behaviors, and social dominance (Dent and Isles 2014). In humans, imprinted gene mutations have been implicated in Prader-Willi and Angelman syndromes (Dykens *et al.* 2011) as well as schizophrenia (Francks *et al.* 2007; Linhoff *et al.* 2009).

## Environmental modifiers of POE

It is likely that many if not all POE are (at least potentially) sensitive to modulation by the external environment. Indeed, it has been postulated that maternal and paternal effects evolved in part to allow parents to transmit environmental cues to their progeny (Mousseau 1998; Uller 2008). And epigenetic variation in general (imprinting being one example) seems to be associated with an environment that is both stressful and predictable (Jablonka and Raz 2009). Moreover, a broad array of environmental perturbations have been found to affect expression of numerous imprinted genes (Kappil *et al.* 2015). In principle, environment-by-POE could arise from exposures both pre- and post-natally: prenatal environment could alter imprinting, uterine tissue, sperm content, etc.; postnatal environment could alter not only imprinting but also maternal or paternal care.

Few studies, however, have explored environment-by-POE on phenotypes related to brain or behavior. Most of the evidence we have reviewed is indirect, usually from experiments designed for a different purpose and lacking the basis for formal environment-by-POE tests. Examples include (all in rodents): pre-gestational maternal stress (Cameron *et al.* 2005) and high fat-diet (Kougias *et al.* 2018) affecting maternal care and, in turn, offspring behavior; maternal exposure to alcohol in a reciprocal cross seeming to induce a POE on behavior (Sittig *et al.* 2011); paternal exposure to cocaine seeming to reduce cerebral volume more than maternal exposure (He *et al.* 2006); exercise plus cognitive training affecting sperm miRNA expression levels and, in turn, behavior (Benito *et al.* 2018).

## Perinatal diet as a potential modifier of behavioral POE

A particularly good lever for investigating environment-by-POE on behavior could be perinatal diet. This is suggested in part by the properties of imprinted genes, namely that: 1) imprinting is believed to largely result from differential allelic methylation and thus to require dietary methyl donors (Crider *et al.* 2012); 2) perinatal dietary restriction has been shown to modulate imprinted gene expression (Harper *et al.* 2014; Kappil *et al.* 2015; Xue *et al.* 2016); and, as described earlier, 3) imprinted genes are known to affect behavior.

Effects of perinatal diet on behavior and DNA methylation have been observed in numerous studies on rodents. Perinatal protein deficiency (PD) and vitamin D deficiency (VDD) both induce methylation changes (Vucetic *et al.* 2010; Lillycrop *et al.* 2007; Kesby *et al.* 2010, 2012) and alter behaviors that model schizophrenia (Burne *et al.* 2004a,b 2006; Palmer *et al.* 2008; Franzek *et al.* 2008; Kesby *et al.* 2006, 2010; Burne *et al.* 2006; Harms *et al.* 2008, 2012; Turner *et al.* 2013; Vucetic *et al.* 2010). Other perinatal diets implying a deficiency in methyl donors have similarly been linked to reduced methylation in the brain (Davison *et al.* 2009; Niculescu *et al.* 2006; Konycheva *et al.* 2011), increased anxiety-like behaviors (Ferguson *et al.* 2005; Konycheva *et al.* 2011), and changes in learning and memory (Konycheva *et al.* 2011; Berrocal-Zaragoza *et al.* 2014). Although such epigenetic effects are not necessarily POE (these studies did not test for POE), given that imprinting is an epigenetic phenomenon they could be. Alternatively, these effects may involve dietary alteration of epigenetic state at loci coding for maternal-specific factors; *i.e.*, maternal effect POE.

In humans, observational studies of the Dutch Hunger Winter (DHW) cohort have found associations between perinatal nutritional deficiency (especially in the late prenatal/early postnatal periods) and increased prevalence of mental illness, along with associated changes in imprinted gene expression (Heijmans *et al.* 2008; Tobi *et al.* 2009).

Yet none of the above studies have shown perinatal diet-modulated POE (hereafter, “diet-by-POE”) on behavior definitively: observed correlative changes in epigenetic state and behavior may be coincidental; paternal environmental exposure might have elicited similar effects as maternal exposure. Indeed, although diet-by-POE has been investigated on non-behavioral traits (Cheverud *et al.* 2011; Haaland *et al.* 2017), to our knowledge, there have been no studies examining diet-by-POE on behavior directly.

## Direct investigation of POE and diet-by-POE on behavior

The above findings motivate an experiment to directly determine the extent of POE and diet-by-POE on behaviors modeling psychiatric disease. If such effects, whether POE or diet-by-POE, can be unambiguously detected, and their genetic architecture characterized, the results could inform the design of an eventual mapping study; our study is a step in that direction. We do this using a relatively neglected but extremely powerful design: we generate a population of female reciprocal F1 hybrids (RF1s) of two classical inbred mouse strains under four different perinatal diets, and then test these RF1s for POE and diet-by-POE on behavior and genome-wide expression.

Our study reveals: 1) POE on behavior and gene expression, many of which are robust to differences in perinatal diet; 2) a possible explanatory pathway connecting imprinting, gene expression, and behavior; and 3) the utility of the RF1 approach as a template for further animal model studies of POE and environment-by-POE on complex traits. To our knowledge, a similar approach in mice has only been performed in Schoenrock *et al.* (2018), where we reciprocally crossed strains of the Collaborative Cross.

## Experimental Materials and Methods

### Reciprocal F1 hybrids as a tool to characterize POE and diet-by-POE

In overview, we investigate POE and diet-by-POE by first generating a population of female reciprocal F1 hybrids (RF1s) of the classical inbred mouse strains C57BL/6J (B6) and NOD/ShiLtJ (NOD) under four different perinatal diets, and then testing for POE and diet-by-POE on behavior and genome-wide expression. Our use of these experimental parameters reflects the following considerations:

1. **Female RF1s** are optimal for detecting POE because they allow the direction of inheritance to be switched while keeping genetic background (save for mitochondria) constant; they also allow unconfounded testing of diet-by-POE when diet is nested within a constant background and direction. By contrast, studies of POE in general (not on behavior) have often used outbred populations (Peripato and Cheverud 2002; Cheverud *et al.* 2011; Lawson *et al.* 2013), which despite some advantages—including the ability to simultaneously map and detect POE, as well as to readily disambiguate between POE from imprinting *vs.* maternal/paternal factors (Hager *et al.* 2008)—cannot hold genetic background constant and are less powerful for POE detection. RF1s have detected POE on behavior in multiple previous studies (Isles *et al.* 2001; Calatayud and Belzung 2001; Calatayud *et al.* 2004; Jiao *et al.* 2013; Shang *et al.* 2017).
2. **Mouse** was chosen for its rapid gestation and development, its versatility as a model for behavioral genetics and environmental perturbation, and the similarity between humans and mice in imprinted gene function (Bartolomei and Ferguson-Smith 2011).
3. **B6 and NOD** inbred strains were selected because i) B6 is the reference genome and is the best characterized strain with respect to behavior; ii) B6 and NOD are among the founder strains for the Collaborative Cross, a population that is an area of focus for our labs; iii) B6 and NOD were both readily available, and B6-NOD crosses generate large litters, facilitating replication; and iv) B6 and NOD are genetically similar enough for a standard B6-expression microarray to be appropriate (Oreper *et al.* 2017), while different enough to potentially elicit POE.

Specifics of the experimental design and analysis are described in this section, the next section, and in Appendices A-J.

### Mice

B6 and NOD mice originated from a colony maintained by Gary Churchill at Jackson Laboratory, and were transferred in 2008 to the FPMdV lab at UNC (this originating colony also produced the G1 breeders of the CC; see Srivastava *et al.* 2017).

**Figure 1:**
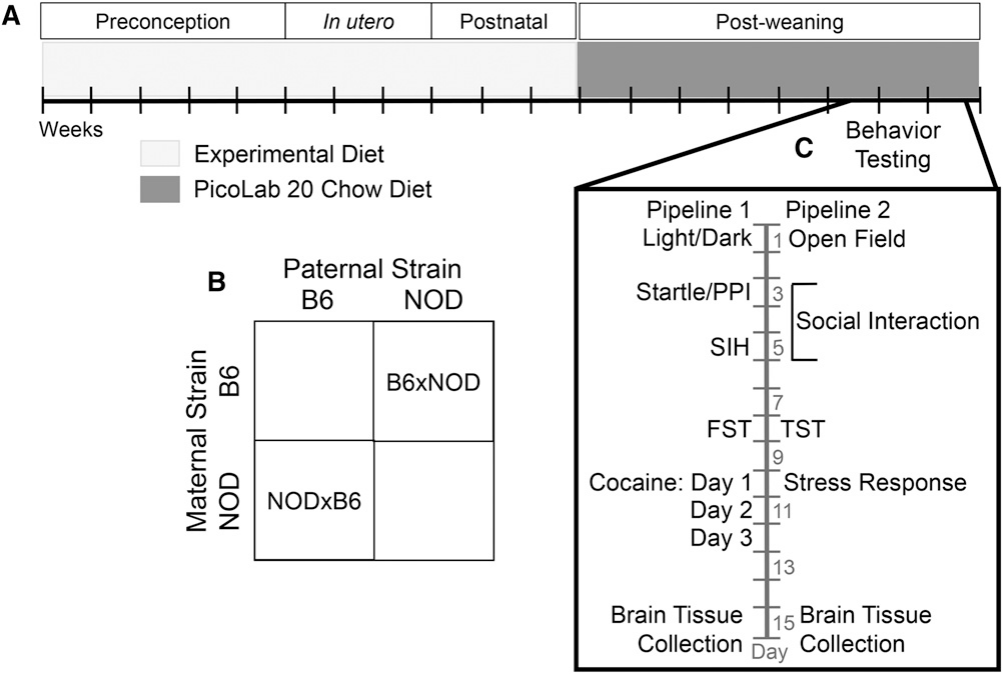
Experimental design to assess POE, perinatal diet, and diet-by-POE on behavior and gene expression in reciprocal F1 hybrids (RF1s). Female NOD/ShiLtJ (NOD) and C57BL/6J (B6) mice were placed on one of 4 experimental diets (protein deficient, vitamin D deficient, methyl enriched, control) at 7 weeks of age (A). After 5 weeks, NOD females were mated to B6 males and B6 females to NOD males generating NODxB6 and B6xNOD RF1 hybrids, respectively (B). Dams remained on their experimental diet throughout gestation and the postnatal period. At PND 21, female F1 hybrids were weaned and placed onto our standard laboratory diet (A). Upon reaching adulthood at PND 60, F1 hybrids were tested in one of two behavioral pipelines. After behavioral testing, mice were killed, and their brain tissue collected for gene expression analysis via microarray and qPCR (C).

Six-week old B6 females (3-8 dams/diet) and NOD females (3-5 dams/diet) were transferred from the FPMdV lab to the Tarantino lab at UNC and acclimated for one week. At 7 weeks of age, dams were placed on one of 5 different experimental diets (described later). At 12 weeks, dams were mated with males of the opposite strain to produce either B6xNOD or NODxB6 F1 hybrids (dam strain listed first; Figure 1B). Sires were removed from the breeding cage once the dam was visibly pregnant. Pregnant dams remained on their experimental diet until litters were weaned, ensuring that offspring were exposed to the experimental diet throughout the entire perinatal period. This study used females from each dam’s first litter exclusively.

At postnatal day (PND) 21: the female, F1 hybrid littermates were weaned into the same cage with 2-5 females/cage (see Table S2 and Table S3 for dam/offspring numbers), and onto a different diet, the post-weaning diet (described later; Figure 1A). F1 hybrids were subsequently bred in one vivarium, but were transferred before testing to a separate behavioral testing vivarium, where they were acclimated for at least one week. Throughout, mice were housed in a specific pathogen free facility on a 12-hour light/dark cycle with lights on at 7 am. All procedures and animal care were approved by the UNC Institutional Animal Care and Use Committee and followed the guidelines set forth by the National Institutes of Health (NIH) Guide for the Care and Use of Laboratory Animals.

### Experimental and post-weaning diets

This study used five experimental diets, administered during the perinatal periods, along with one additional diet administered post-weaning. The experimental diets (from Dyets Inc., Bethlehem, PA) were as follows: vitamin D deficient (VDD; #119266), protein deficient (PD; 7.5% casein; #102787), methyl donor deficient (MDD; #518892), methyl donor enriched (ME; #518893), and standard (Std; #AIN-93G). The VDD and PD diets were nutritionally matched to the Std diet, whereas the MDD and ME diets were matched to each other. Nutrient compositions for each experimental diet are listed in Table S1. Experimental diets continued for 3 weeks after birth, to capture early postnatal development in mice that mirrors late prenatal development in humans (Clancy *et al.* 2007; Workman *et al.* 2013).

At three weeks old, all pups were weaned onto the post-weaning diet, PicoLab Rodent Chow 20 (Pico rodent chow 20; Purina, St. Louis, MO, USA), which was the standard UNC laboratory diet (composition described at https://www.labdiet.com/cs/groups/lolweb/@labdiet/documents/web_content/mdrf/mdi4/∼edisp/ducm04_028436.pdf). Food and water were available *ad libitum* throughout the experiment.

### Behavior assays

To ensure a standardized genetic background that included the sex chromosomes, the only tested F1 hybrids were female. Mice were 61.7 days old ± 2.6 SD at the onset of testing. All behavioral testing was performed during the light part of the light/dark cycle between 8:00 am and 12:00 pm.

Mice were placed into one of two behavioral testing pipelines (Figure 1C) to assess anxiety- and depressive-like behavior, stress response, sensorimotor gating, and response to a psychostimulant: Pipeline 1—the light/dark assay, startle/prepulse inhibition (PPI), stress-induced hyperthermia (SIH), forced swim test (FST) and cocaine response (N = 91); or Pipeline 2—open field (OF), social interaction test, tail suspension and restraint stress (N = 87). We chose the tests for each pipeline to try to cover as wide a range of endophenotypes as possible. In total, 34 behavioral measures were collected, with 22 in pipeline 1 and 12 in pipeline 2 (Table 1). Each cage of animals was randomly assigned to one of the two pipelines; all animals in the same cage—littermates—were tested in the same (and only in a single) pipeline.

**Table 1:** POE, perinatal diet effect, and diet-by-POE on behavioral phenotypes. For each phenotype, the table shows the modeled variables, along with their corresponding q-values (FDR), which account for multiple testing within a behavioral pipeline. Significant/suggestive q-values are bolded. †q < 0.2 (suggestive), *q < 0.05, **0.01, ***0.001 respectively. POE = parent of origin effect; PPI = prepulse inhibition; CORT = corticosterone; SIH-T1 = basal temperature; SIH-T2 = post-stress temperature; SIH-delta = (T2-T1). For completeness, p-values before multiple testing correction are included as well, but they are not directly used to test significance

Pipeline	Test	Phenotype	Covariates	p value			q value		
				POE	Diet	Diet x POE	POE	Diet	Diet x POE
**1**	Light/Dark	Total Distance	Batch, Dam	0.0493	0.481	0.99	**0.181**†	0.814	0.99
		Distance Dark		0.0187	0.646	0.985	**0.103**†	0.836	0.99
		Distance Light		0.273	0.247	0.905	0.43	0.68	0.99
		% Time Dark		0.373	0.175	0.392	0.547	0.561	0.92
		% Time Light		0.226	0.129	0.341	0.414	0.561	0.92
		Total Transitions		0.0772	0.904	0.61	0.243	0.904	0.92
	Startle/Prepulse Inhibition	AS50 Average	Batch, Chamber, Dam	0.399	0.617	0.0904	0.548	0.836	0.731
		AS50 Latency		0.935	0.149	0.432	0.98	0.561	0.92
		Average PPI 74	Batch, Chamber, Dam, Pup	0.217	0.481	0.565	0.414	0.814	0.92
		Average PPI 78		0.22	0.0714	0.636	0.414	0.524	0.92
		Average PPI 82		0.0307	0.00274	0.445	**0.135**†	**0.0595**†	0.92
		Average PPI 86		0.123	0.179	0.669	0.301	0.561	0.92
		Average PPI 90		0.988	0.62	0.0997	0.988	0.836	0.731
	Stress-Induced Hyperthermia	SIH-T1	Batch, Test Order, Dam	0.273	0.828	0.61	0.43	0.904	0.92
		SIH-T2		0.000887	0.628	0.0624	0.00975**	0.836	0.731
		SIH-Delta		0.648	0.879	0.473	0.839	0.904	0.92
	Forced Swim	% Immobility	Batch, Arena, Dam	0.111	0.317	0.531	0.301	0.776	0.92
	Cocaine Response	Day1 Distance	Batch, Dam	0.000671	0.43	0.332	0.00975**	0.814	0.92
		Day2 Distance		0.00221	0.47	0.325	0.0162*	0.814	0.92
		Day3 Distance		0.782	0.692	0.876	0.906	0.846	0.99
		Day3-Day2 Distance		0.73	0.771	0.897	0.892	0.893	0.99
	Body Weight	Body Weight	Batch, Dam	0.908	0.00541	0.913	0.98	**0.0595**†	0.99
	**2**	Open Field	Distance Moved	Batch, Dam	0.013	0.647	0.555	**0.156**†	0.647	0.832
			% Center Time		0.319	0.234	0.0144	0.638	0.592	**0.172**†
			Average Velocity		0.428	0.128	0.145	0.638	0.511	0.435
			Jump Counts		0.788	0.312	0.223	0.788	0.592	0.447
			Vertical Counts		0.0763	0.103	0.932	0.318	0.511	0.932
Boli Count			0.466		0.113	0.301	0.638	0.511	0.517
Social Interaction		% Time Stranger	Batch, Stranger Box, Dam	0.425	0.493	0.182	0.638	0.592	0.438
		Transitions		0.705	0.633	0.72	0.769	0.647	0.864
Tail Suspension		% Immobility	Batch, Dam	0.536	0.305	0.652	0.643	0.592	0.864
Restraint Stress		Basal CORT	Batch, Test Order, Dam	0.478	0.475	0.923	0.638	0.592	0.932
		10 min CORT		0.0796	0.372	0.0735	0.318	0.592	0.388
		A CORT		0.113	0.412	0.097	0.338	0.592	0.388

Both testing pipelines were applied to mice from 3 separate breeding batches, over 3 months (to accommodate the capacity of our animal facility). To avoid confounding, each breeding batch included offspring from both RF1 directions, as well as from at least 2 diet exposures. For each diet and RF1 direction, we tested litters from at least 2 dams (N = 4 ±1.4; see Table S2 for dam and offspring counts). One mouse in the NODxB6 ME group was killed due to injury on the day of social interaction testing; there is no data for this mouse for social interaction or for any subsequent test. There is no restraint stress data for another 4 mice (1 NODxB6 ME, 2 B6xNOD Std, 1 B6xNOD VDD), due to either death in the restrainer or insufficient serum collected for radioimmunoassay (RIA) analysis of corticosterone (CORT) levels.

#### Open field (OF):

Mice were placed in the OF arena for 10 min. The OF apparatus (ENV-515-16, Med Associates, St. Albans, VT, USA) was a 43.2x43.2x33 cm arena, consisting of a white Plexiglas floor and clear Plexiglas walls with infrared detection beams at 2.54 cm. intervals on the x, y, and z axes that automatically tracked mouse position and activity throughout each experimental session. The apparatus was in a sound-attenuating chamber (73.5x59x59 cm) fitted with two overhead light fixtures containing 28-V lamps. Mice were placed in the OF arena for 10 min. The OF apparatus (ENV-515-16, Med Associates, St. Albans, VT, USA) was a 43.2x43.2x33 cm arena, consisting of a white Plexiglas floor and clear Plexiglas walls with infrared detection beams at 2.54 cm. intervals on the x, y, and z axes that automatically tracked mouse position and activity throughout each experimental session. The apparatus was in a sound-attenuating chamber (73.5x59x59 cm) fitted with two overhead light fixtures containing 28-V lamps. Mice were scored for total distance traveled (cm), average velocity (cm/s), number of vertical movements (rearing), and percent time spent in the center of the arena (a 22.86 cm^2^ central part of the arena). These measurements were recorded in 5 bins of 2-minute width, and were scored in post-session analyses using Activity Monitor 5.1 software (Med Associates). The testing apparatus was cleaned with a 0.25% bleach solution between test subjects.

#### Social interaction:

Social approach was measured in a 3-chamber social interaction apparatus during a 20-minute test [as per Moy *et al.* (2007)]. Briefly, the first 10 min was a habituation period in which the test mouse was given free access to all 3 chambers. The number of times the mouse moved from one chamber into another chamber during the first 10-min period was recorded as ‘total number of transitions. During the second 10 min, the test mouse was given a choice between two chambers: i) one chamber containing an empty circular mesh enclosure; ii) a second chamber containing the same mesh enclosure, but holding a stranger mouse—specifically a B6 female which had previously been habituated to the social chamber, mesh enclosure, and other female mice. The amount of time the test mouse spent in the chamber with the stranger mouse was recorded and is reported as “percent stranger time”, a measure of social preference. The stranger mouse was not tracked in the social interaction tests.

#### Tail suspension:

Mice were suspended by a piece of laboratory tape wrapped around the tail and hung from a hook at the top of a 24.13 cm × 17.78 cm × 17.78 cm white acrylic enclosure. Mice were videotaped for the entire 4-minute session, and videotapes were analyzed for immobility during the last 2 min using the Actimetrics Freeze Frame analysis program (Actimetrics, Wilmette, IL). Percent immobility during the last two minutes is reported as a measure of depressive-like behavior (Miller *et al.* 2010).

#### Restraint stress:

Restraint was used to elicit a stress response that was then quantified by measurement of CORT levels in the serum. A retro-orbital blood sample was taken immediately prior to placing the mice into a Broome-Style restraint tube (Plas Labs, Inc., Lansing, MI, USA) for 10 min. Immediately upon removal from the restrainer, a second retro-orbital eye bleed was performed. Whole blood was centrifuged to isolate serum, and then the CORT levels were measured by competitive RIA per the manufacturer’s protocol (MP Biomedicals, Santa Ana, CA, USA).

#### Light/dark:

The open field arena described above was converted to a light dark apparatus by placement of an opaque polycarbonate black box that occupied one third of the arena space, thus allowing the mouse to choose between the light or dark part of the apparatus. Mice were placed in the lighted area immediately adjacent to and facing the entry to the dark enclosure and remained in the apparatus for 10 min. The amount of time (sec), distance moved (cm) and number of transitions between the dark and light zones was scored in 5-minute bins in post-session analyses using Activity Monitor 5.1 software (Med Associates). The testing apparatus was cleaned with a 0.25% bleach solution between test subjects.

#### Startle and prepulse inhibition (PPI):

Acoustic startle and PPI of the startle response were both measured using the San Diego Instruments SR-Lab system (San Diego, CA), and following the protocol in Moy *et al.* (2012). Mice were placed in a Plexiglas cylinder located in a sound-attenuating chamber that included a ceiling light, fan, and a loudspeaker that produced the acoustic stimuli (bursts of white noise). Background sound levels (70 dB) and calibration of the acoustic stimuli were confirmed with a digital sound level meter. Each test session consisted of 42 trials, presented following a 5-min habituation period. There were 7 types of trials: no-stimulus trials, trials with a 120 dB acoustic startle stimulus (a.k.a., ASR), and 5 trials in which a 20 ms prepulse stimulus (74, 78, 82, 86, or 90 dB) was presented 100 ms before the onset of the 120 dB startle stimulus. The different trial types were presented in 6 blocks of 7, in randomized order within each block, with an average intertrial interval of 15 sec (range: 10 to 20 s). Measures were taken of the startle response amplitude (RA) for each trial, defined as the peak response recorded from the onset of startle stimulus to the end of the 65-msec sampling. The PPI for each prepulse sound level was calculated as:

$$\text{PPI}=100-\left[\frac{\text{RA with prepulse \& startle stimulus}}{\text{RA with only startle stimulus}}\right]\times 100$$

#### Stress-induced hyperthermia (SIH):

Each tested mouse was individually removed from its home cage, and then its body temperature (T1) was measured. Specifically, a lubricated digital thermometer probe was inserted 1-1.5 cm into the rectum for approximately 10 sec. The mouse was then returned to its home cage, and 10 min later the temperature measurement was repeated (T2). The difference in body temperature, ΔT=T2−T1, was used as a measure of anxiety-like behavior (Adriaan Bouwknecht *et al.* 2007). Basal temperature was measured for all mice within a single cage in under a minute, to avoid increases in body temperature due to anticipatory stress.

#### Forced swim test (FST):

Mice were placed in a glass-polycarbonate cylinder (46cm tall X 21cm in diameter) filled with water (25-28°) to a depth of 15 cm for 6 min. The duration of immobility during the last 4 min of the test period was scored using Ethovision 7.0 automated tracking software (Noldus, Leesburg, VA). Immobility was defined as no movements other than those needed to stay afloat. Mice were monitored continuously, and removed if they were unable to keep their nose or heads above water for more than 30 sec. Percent immobility was reported as a measure of depressive-like behavior (Porsolt *et al.* 1977).

#### Cocaine-induced locomotor activation:

Cocaine-induced locomotor activity was measured over a 3-day test protocol in the OF arena described above. On days 1 and 2, mice were given an intraperitoneal injection of saline before being placed into the OF arena for 30 min, and then returned to their home cage. Total distance moved on Day 1 and Day 2 was used to measure novelty-induced and baseline locomotor activity, respectively.

The Day 3 protocol was nearly identical, but instead of saline, mice were injected with 20 mg/kg cocaine (Cocaine HCl; Sigma-Aldrich, St. Louis, MO). The difference between total distance traveled on Day 3 and Day 2 was used to measure cocaine-induced locomotor activation.

#### Body weight:

Adult body weight was recorded for mice in pipeline 1 prior to startle/PPI and cocaine administration.

### Gene expression

To identify genes subject to POE and/or perinatal-diet effect, whole-brain expression was measured by microarray, and key expression results were later validated with qPCR.

#### Tissue extraction:

Three days after completion of behavioral testing (at an average age of 76.7 ± 2.6 days), mice were killed, and whole brain was removed. The cerebellum was discarded, and the cerebral portion was kept and hemisected into the left and right hemispheres. Each brain hemisphere was placed into a separate tube and immediately flash frozen in liquid hydrogen. Right brain hemispheres were pulverized using a BioPulverizer unit (BioSpec Products, Bartlesville, OK). Pulverization batches were designed to prevent contamination between mice from different crosses or diets.

#### RNA extraction:

Total RNA was extracted from 25mg of pulverized right brain hemisphere tissue, using an automated bead-based capture technology (Maxwell 16 Tissue LEV Total RNA Purification Kit, AS1220; Promega, Madison, WI). Purified mRNA was evaluated for quality and quantity by the Nanodrop Spectrophotometer (Thermo Scientific) and the Bioanalyzer 2100 (Agilent).

#### Microarray expression measurement:

Of the 178 behaviorally-phenotyped, female B6xNOD and NODxB6 F1s, 96 females were selected for microarray measurement of gene expression. The choice of 96 mice was balanced to include both directions of reciprocal cross offspring, all 4 diets, as well as both behavioral test pipelines, while simultaneously maximizing the number of represented litters. Gene expression was measured using the Affymetrix Mouse Gene 1.1 ST Array. All samples were processed by the Functional Genomics Core at UNC.

#### qPCR expression measurement:

Commercially available Taqman qPCR assays for *Carmil1* (Life Technologies, Mm01158156_m1) and *Meg3* (Life Technologies, Mm00522599_m1) were used to estimate gene expression levels. Specific primers were chosen to ensure that there was no difference between NOD and B6 in the primer binding region. For each sample, mRNA was retro-transcribed to cDNA using 200ng of starting RNA (SuperScript III First-Strand Synthesis System, 18080051; Thermo Fisher Scientific, Waltham, MA) following the manufacturer’s protocol. The amplification curve was calibrated using an *Rfng* (Life Technologies, Mm00485703_m1) reference assay. All assays were performed following the manufacturer’s protocol on an ABI StepOne Plus Real-Time PCR System (Life Technologies, Carlsbad, CA), and in duplicate; each sample was assayed on 2 of 3 available plates. Samples were plated such that breeding batch, which explained much of the microarray expression variance, was partially confounded with qPCR plate. Cycle thresholds were determined using ABI CopyCaller v2.0 software on default settings. All available brain samples were assayed, regardless of hemisphere.

## Computational and Statistical Methods

### Statistical analysis of behavior

Diet effects, POE, and diet-by-POE on behavior were evaluated using a linear mixed model (LMM). The LMM was mostly similar between behaviors (distinctions below) and always included a fixed effect covariate of breeding batch, a random effect of dam, and fixed effects of diet, POE and diet-by-POE, with these last three being the subject of significance tests based on their addition to the model in that order (Appendix A). To satisfy parametric assumptions, tests were performed after each behavior had been transformed to approximate residual normality (Appendix B).

Behavior-specific elaborations were as follows. Fixed effect covariates were added for: 1) the swimming chamber for FST, 2) testing order for SIH and restraint stress, and 3) the box holding the stranger mouse for the social interaction test. Random effects were added for the repeated measures phenotypes startle and PPI, namely representing pup and chamber (Appendix A). For ASR data, the modeled outcome was the raw ASR divided by the mouse body weight. For the PPI at each prepulse intensity, the modeled outcome was the average PPI response divided by the weight-adjusted ASR value.

LMMs were fit in R (R Core Team 2016) using lme4 (Bates *et al.* 2015) and *p*-values calculated by a type I (*i.e.*, sequential) sum of squares ANOVA using Satterthwaite’s approximation implemented in lmerTest (Kuznetsova *et al.* 2015). To account for multiple testing, the *p*-values were pooled over all behaviors in each pipeline, but separated per effect type (diet effects, POE, diet-by-POE); then, each pipeline/effect type combination was subject to a Benjamini-Hochberg false discovery rate correction, generating q-values (Benjamini and Hochberg 1995).

### Microarray preprocessing

Microarray probe alignments to the GRCm38.75 C57BL/B6J reference genome (the reference we use throughout) were used to infer probe binding locations (Appendix G). Using these locations, along with Affymetrix Power Tools (APT) 1.18 software (Affymetrix 2017), probes and probesets at biased/uninformative binding locations (Appendix H) were masked. Masking reduced the original set of 28,440 non-control probesets to only 20,099 probesets (representing 19,224 unique genes, including X chromosome genes). For the remaining probesets, RMA (Irizarry *et al.* 2003) was applied to the non-masked probes to compute a probeset expression score per probeset. Each probeset’s position was defined as the binding location of its first non-masked probe. The expression of one mouse was inadvertently measured twice; these probeset measurements were pairwise averaged.

### Statistical analysis of gene expression

Data from 95 microarray-assayed mice and 20,099 probesets was used to test diet effects, POE, and diet-by-POE on gene expression as follows. For each probeset: 1) fixed-effect nuisance covariates were regressed out of the expression score to generate adjusted expression values; 2) the adjusted expression was transformed to ensure residual normality; 3) the resulting values were significance tested for diet, POE and diet-by-POE, built up sequentially in that order, in an LMM that accounted for dam (using the R package nlme Pinheiro *et al.* 2016).

The covariates regressed out of the expression scores included both known experimental factors (breeding batch, pipeline) and latent factors estimated from the data, the latter defined using a modified form of Supervised Surrogate Variable Analysis (Leek 2014) that was adapted to accommodate random effects (Appendix D).

The distribution of (nominal) p-values for each effect type appeared to be inflated. To help correct for this, p-values were adjusted by a genomic control-like procedure (Dadd *et al.* 2009) whereby, for all p-values within an effect-type, an inflation factor was estimated and then divided out (Appendix E). Then, to control for multiple testing, and per effect type, we applied family-wise error rate (FWER) control using a bespoke permutation procedure that made minimal parametric assumptions while accounting for between-probeset correlations (Appendix F); this was preferred over an FDR correction owing to the latter’s strong reliance on nominal p-values being valid and unbiased.

### Analysis of imprinting status

Each microarray probeset was classified as measuring imprinted gene expression if its probe sequences either: 1) hybridized to the sequence of an imprinted gene identified in MouseBook (Blake *et al.* 2010) or in Crowley *et al.* (2015); or 2) hybridized within 100bp of these known imprinted genes. All together, 241 probesets were classified as measuring imprinted regions, corresponding to 182 unique imprinted genes. Each probeset was also categorized as to whether it revealed a significant (q-value < 0.05) POE on expression of the probed region. The association between imprinting status and significant expression POE was tested using Fisher’s exact test, which was also used to test the association (restricted to imprinted genes) between genes exhibiting POE in our dataset those exhibiting POE in Crowley *et al.* (2015).

### Analysis of qPCR validation data

An apparent POE on microarray expression of *Carmil1* and a diet-by-POE on *Meg3* were validated by analysis of their respective qPCR data as follows. Each gene’s qPCR relative-cycle-threshold (relative to *Rfng*, Appendix I) was transformed for residual normality, and then modeled by an LMM that accounted for pipeline, the interaction of breeding batch with qPCR plate (as a random effect), and dam (random effect), as well as the diet, POE, and diet-by-POE effects. LMMs were fit using lme4 (Bates *et al.* 2015), with p-values computed using lmerTest (Kuznetsova *et al.* 2015). qPCR data analysis was repeated in three sets of mice: 1) 85 mice assayed by both microarray and qPCR; 2) 30 mice newly assayed by qPCR alone; and 3) all 115 qPCR’d mice.

### Mediation analysis

POE were observed upon several behaviors, as well as upon the expression of the non-imprinted gene, *Carmil1*. To identify (potentially imprinted) genes exerting POE on these outcomes, we performed a mediation analysis (Hayes 2009) genome-wide. That is, for each outcome above, and for each potential mediator gene, we tested whether the gene’s expression mediated POE on the outcome (details in Appendix J). For completeness, and to generate percentile-based significance thresholds, we tested every gene as a candidate mediator whether or not we observed POE on the candidate in mediation-free analysis.

This test was performed using a model (see Figure 8 notation) in which the outcome was the sum of: 1) outcome-specific nuisance effects (which also affect the candidate mediator gene); 2) a diet-specific *direct* effect of parent-of-origin $\left(c_{d}^{\prime }\right),$ and 3) a diet-specific *indirect* effect of parent-of-origin, that is mediated by way of POE on the candidate mediator gene’s expression $\left(a_{d}b\right).$ Candidate mediator genes with a significant *average* indirect effect $\left(ab=\bar{a_{d}}b\right)$ on POE were identified as true mediators. Candidate mediator genes for which the indirect and direct effect had opposite signs were further classified as suppressors.

We note that in this model, diet does *not* modulate the effect of mediator expression on outcome; the indirect effect is diet-specific only insofar as diet affects mediator expression.

#### Mediation analyzed using a Bayesian approach, and Minimum Tail Probability:

Most simple mediation analyses are handled using frequentist methods. However, our mediation model required that we estimate an indirect effect across multiple diets, all while accounting for the random effect for dam. For this type of complexity, a Markov Chain Monte Carlo (MCMC)-based Bayesian approach was ideal, providing the necessary flexibility to easily provide point and interval estimates of the indirect effect, all without the need to derive an analytic form (Yuan and MacKinnon 2009; Wang and Preacher 2015). Our mediation model, described in more detail in Appendix J, was implemented in JAGS [Just Another Gibbs Sampler; Plummer (2003, 2016)]. Posterior medians and credible intervals for direct and indirect effects were estimated from Gibbs samples. To obtain a measure of “mediation significance”, we estimated the indirect effect’s “Minimum Tail Probability” (MTP): the minimum of the sample-based, upper and lower tail probabilities of the indirect effect, where we deemed MTP ≤.05 significant (as used in, *e.g.*, Schoenrock *et al.* 2016).

#### Mediation of Carmil1 expression:

Mediation modeling of the *Carmil1* expression outcome was restricted to data from mice in which expression was measured. Breeding batch, pipeline, and dam (a random effect), were modeled as nuisance effects acting on both the mediator gene and on *Carmil1*.

#### Mediation of behavior:

All behavior outcomes were tested for gene mediation of POE, whether or not expression-free analysis had revealed POE on that outcome. Modeling was restricted to data from mice in which expression and behavior were both measured. Dam, breeding batch, and behavior-specific covariates were modeled as nuisance effects on both mediator and outcome. Pipeline was *not* modeled, as each behavior was only measured in one pipeline. For PPI outcomes, groups of measurements from the same mouse/prepulse intensity were averaged together into a single value.

#### Aggregate mediation of behavior:

To quantify each gene’s aggregate level of mediation over *all* behaviors, we defined a statistic inspired by the Fisher combined p-value (Fisher 1925): the “Combined Tail Probability” (CTP; Appendix J), an aggregate of MTPs over all behaviors. Aggregate levels of mediation were also assessed by counting how often a given mediator was among the 3 most significant mediators for any behavior.

### Reporting significant genes *vs.* probesets

The number of genes we report as significantly affected by some factor (*e.g.*, diet) is generally not equal to the number of significantly affected probeset measurements. The mismatch arises because some genes (*e.g.*, *Snord 115*) are assayed by more than one probeset, and some probesets simultaneously assay more than one gene (*e.g.*, overlapping genes). For each significantly affected multi-gene probeset, we propagate significance to all of its assayed genes; every gene covered by a significantly affected probeset is itself considered significantly affected.

### Test for miRNA regulation of significantly affected genes

To evaluate the validity of the diet-by-POE on *Mir341*, we tested whether the set of non-*Mir341* genes subject to diet-by-POE was enriched for *Mir341*’s predicted targets of regulation. Specifically, we used miRHub (Baran-Gale *et al.* 2013), allowing it to consider all miRNA targets predicted by TargetScan (Agarwal *et al.* 2015), regardless of whether those targets were conserved in another species.

### Segregating variant determination

Variants segregating between NOD and B6 with >.95 probability were identified using ISVdb (Oreper *et al.* 2017).

### Computational resources

Computation was performed on Longleaf, a slurm-based cluster at UNC. Up to 400 jobs were run at a time in parallel. Computation was completed in approximately 6 days.

### Data Availability

Data and supplemental results files are stored on Zenodo at https://doi.org/10.5281/zenodo.1168577 (Oreper *et al.* 2018). File S1 contains detailed descriptions of all supplemental files. File S2 contains chromosome sizes. File S3 contains exon data. File S4 contains Snord data. File S5 contains imprinted genes from Crowley *et al.* (2015), MouseBook, and the union thereof. File S6 contains NOD variants. File S7 contains covariates for RF1s and their dams. File S8 contains Affymetrix library files for the Exon 1.1 ST and 1.0 ST microarrays. File S9 contains Exon 1.0 ST probe binding locations. File S10 contains raw (CEL) microarray-measured expression for RF1s, as well as processed and transformed expression matrices. File S11 contains RF1 behavior and weight data, including: weights, cocaine response light/dark, OF, restraint stress, SIH, sociability, startle/PPI, and tail suspension data, and includes tranformed versions of phenotypes. File S12 contains behavior models for mediation analysis. File S13 contains pulverized brain data. File S14 contains qPCR data, including transformed qPCR phenotypes. File S15 contains MPD datasets, including descriptions, and including transformed versions. File S16 contains full results of models that incorporate microarray expression, including mediation analysis. File S17 contains results of sex-ratio modeling. Code to generate results is available at https://github.com/danoreper/mnp2018.git.

## Results

### Overview and key results

NOD and B6 mice were reciprocally crossed, with F1 hybrids exposed perinatally to Std, VDD, ME, PD, and MDD diets. Of these, the MDD diet was eventually dropped due to a near total lack of reproductive/weaning productivity (Table S2; see also **Supplemental Methods and Results**). Following weaning, the female F1 hybrids were tested in one of two different pipelines, each of which consisted of a different set of behavioral tests (Figure S2). Following behavioral testing, whole brain gene expression was measured via microarray. Analysis and validation led to the following key results (detailed in subsequent subsections):

- Parent-of-origin significantly affected 3 behaviors, including multiple locomotor behaviors and SIH behavior. Data were suggestive of POE on 4 additional behaviors.
- Perinatal diet had a suggestive (but non-significant) affect on body weight and PPI behavior.
- Diet-by-POE had a suggestive effect on OF percent center time.
- Diet, POE, and diet-by-POE significantly acted on expression of 37, 15, and 16 genes respectively.
- The significance of diet’s effect on expression was primarily driven by ME.
- Notable POE were observed on *Snord 115*, *Airn*, and most significantly on *Carmil1*, a non-imprinted gene.
- POE on *Carmil1* was qPCR-validated in two sets of mice: the microarrayed mice, and a new set of mice.
- The set of genes significantly affected by POE is enriched for genes that have been previously shown to be imprinted/near an imprinted gene.
- POE on *Carmil1* may be mediated (specifically, suppressed) by the expression of the imprinted gene *Airn*;
- *Carmil1*, and *Snord 115*, and especially *Airn* seem to mediate POE on multiple behaviors. These, along with other identified mediators of behavioral POE, tend to be suppressors.

### Effects on behavior

Post-FDR correction, POE, diet, and diet-by-POE significantly affected 3, 0, and 0 behaviors, respectively (where “significant” effects are those with a q-value less than  α=.05). Data were suggestive of another 4, 2, and 1 behaviors affected by POE, diet, and diet-by-POE, respectively (where “suggestive” effects are those with a q-value less than α=.2). Table 1 shows the per-variable q-values; Table S5 shows Tukey p-values for variable level contrasts.

#### POE acts upon several locomotor behaviors:

**Figure 2:**
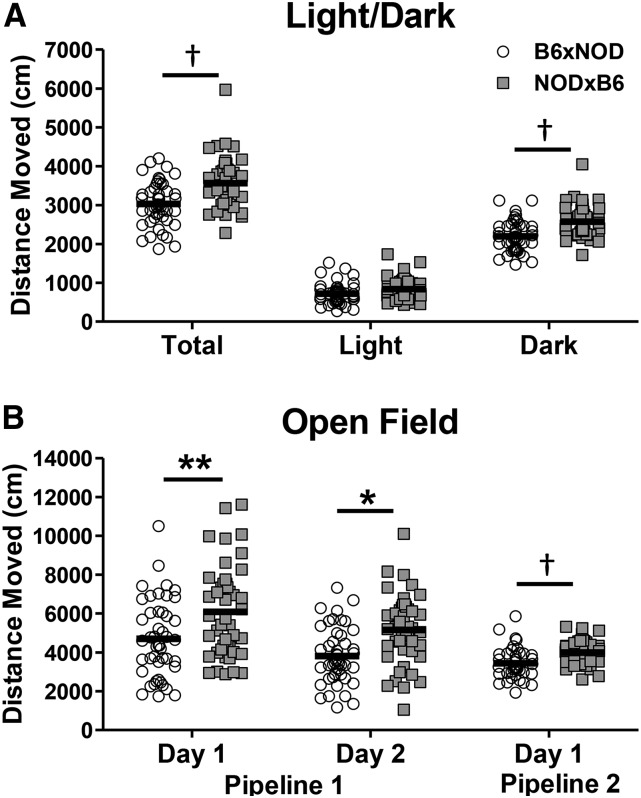
POE on locomotor behavior are consistent across behavioral tests and pipelines. (A) Light side, dark side, and total distance moved in the light/dark arena for individual B6xNOD (n = 46) and NODxB6 (n = 45) mice (bars indicate mean). (B) OF distance moved for B6xNOD (n = 46) and NODxB6 (n = 45) mice, in Pipeline 1 on Day 1 and 2 of a 30 min cocaine response test when the mice received an ip saline injection; Distance moved in Pipeline 2 in a separate 10 min OF test (B6xNOD:n = 39, NODxB6:n = 48). For all assays, NODxB6 mice move significantly more than B6xNOD mice. †q < 0.2 (suggestive), *q < 0.05, **q < 0.01.

Across several assays and both pipelines, a significant or suggestive POE was observed on 5 different assessments of locomotor behavior. Specifically, in pipeline 1, in the Light/Dark test, a suggestive POE was observed on both total distance and distance moved on the dark side of the arena (q = 0.181, q = 0.103, respectively) but not on light side distance (q = 0.43; Figure 2A). Also in pipeline 1, in the cocaine response assay, a POE was observed on total OF distance on both the baseline and the habituation day (Day 1, q = 0.00975; Day 2, q = 0.0162; respectively) (Figure 2B). In pipeline 2, in a separate set of OF-assessed mice, data were suggestive of POE upon total-distance moved (q = 0.156; (Figure 2B).

In all 5 locomotor behaviors that were significant or suggestive of POE, NODxB6 mice moved more than B6xNOD mice. This pattern, coupled with results from previous studies that show increased activity of NOD over B6 (**Supplemental Methods and Results**), suggests that in terms of activity, our hybrids are more similar to the maternal than the paternal line.

#### POE acts upon SIH and PPI:

**Figure 3:**
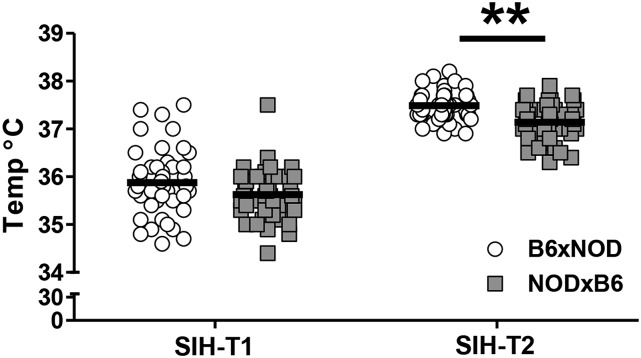
POE on baseline (SIH-T1) and post-stress induced temperature (SIH-T2) in the stress induced hyperthermia test. Data are for individual B6xNOD (n = 46) and NODxB6 (n = 45) mice (bars indicate mean). For SIH-T2 B6xNOD mice have higher temperature than NODxB6 mice. A similar, though non-significant pattern seems to occur in the the SIH-T1 data.

POE was also observed on post-stress temperature in the SIH assay, with B6xNOD mice having higher temperatures (q = 0.00975; Figure 3). Non-significant/non-suggestive effects, but in the same direction, were also seen for both basal temperature (SIH-T1) and change in temperature (SIH-delta), consistent with a small difference in basal temperature being magnified after stress. A suggestive POE was also observed on PPI at 82 decibels, with B6xNOD mice exhibiting a higher percent PPI than NODxB6 (q = 0.156; Figure S3A).

#### Diet may affect body weight and PPI:

**Figure 4:**
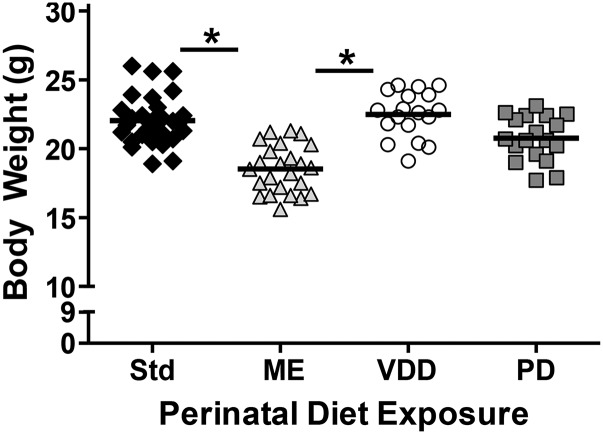
Effect of perinatal diet exposure on body weight in adulthood. Body weight of individual mice (bars indicate mean) exposed to either a control (Std, n = 31), methyl enriched (ME, n = 24), protein deficient (PD, n = 18) or vitamin D deficient (VDD, n = 18) diet during the perinatal period. Perinatal diet had a suggestive effect on body weight (q = 0.0595). In testing individual contrasts (without multiple testing correction), * indicates a difference between ME from Std and VDD mice (*P* < 0.05).

Perinatal diet had a suggestive effect on body weight (q = 0.0595, Figure 4), with mice exposed to ME diet weighing less than mice exposed to Std and VDD diets (Tukey post-hoc *P* = 0.0228 and *P* = 0.0402). Diet also had a suggestive effect on sensorimotor gating: in particular, PPI at 82 decibels (q = 0.0595; Figure S3B). At 78 decibels, PD had a non-significant/non-suggestive (q =0.524), but similar effect (Figure S3B). At both 78 and 82 decibels, PPI seemed greatest for PD mice compared to other diets, although individual contrasts were not significant (Figure S3B, Table S5).

#### Diet may interact with parent-of-origin to alter percent center time:

**Figure 5:**
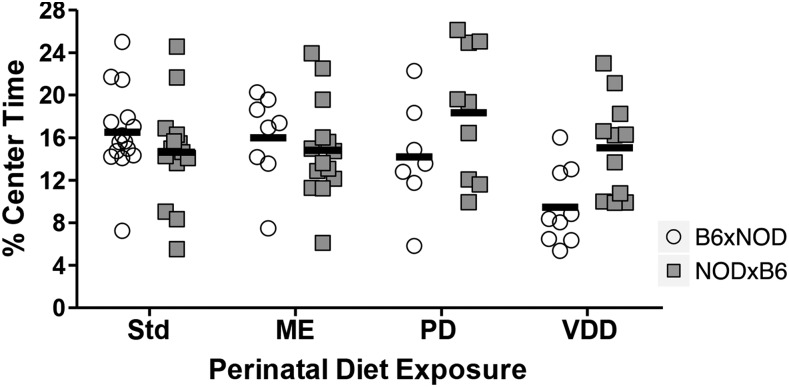
Perinatal diet-by-POE on percent center time in the 10 min OF test, for B6xNOD and NODxB6 mice exposed to Std (n = 15,14), ME (n = 8,14), PD (n = 7,9) or VDD (n = 9,11) diets; data are suggestive of diet-by-POE (q = 0.172).

A suggestive diet-by-POE was observed on percent center time in the OF test (q = 0.172; Figure 5). In this test, NODxB6 mice exposed to VDD and PD diets spent more time in the center of the arena than diet-matching B6xNOD mice, but no such difference was seen for ME or Std diets. Similar but non-significant/non-suggestive effects were seen on OF locomotor activity (Figure S4).

### Effects on whole-brain gene expression

Gene expression at each microarray probeset was tested for POE, diet effects, and diet-by-POE. Significance was assessed using a permutation-based family wise error rate (FWER) threshold. See Table 2 for a summary of expression results.

**Table 2:** Microarray-measured effects on expression. For each effect type, the table specifies the significance threshold value, as well as the number of probesets, genes, and imprinted genes whose expression was significantly affected. Note that: i) some probesets measure multiple genes, and some genes are measured by multiple probesets; ii) imprinting is enriched among genes subject to POE, and iii) diet does not affect any imprinted gene, whereas one imprinted gene is subject to diet-by-POE

Effect Type	-log_10_(FWER thresh)	# Significantly affected		
		Probesets	Genes	Imprinted genes
POE	5.08	20	15	9
Diet	4.97	33	37	0
Diet x POE	4.68	17	16	1

#### POE detected on 15 genes, 9 imprinted:

**Figure 6:**
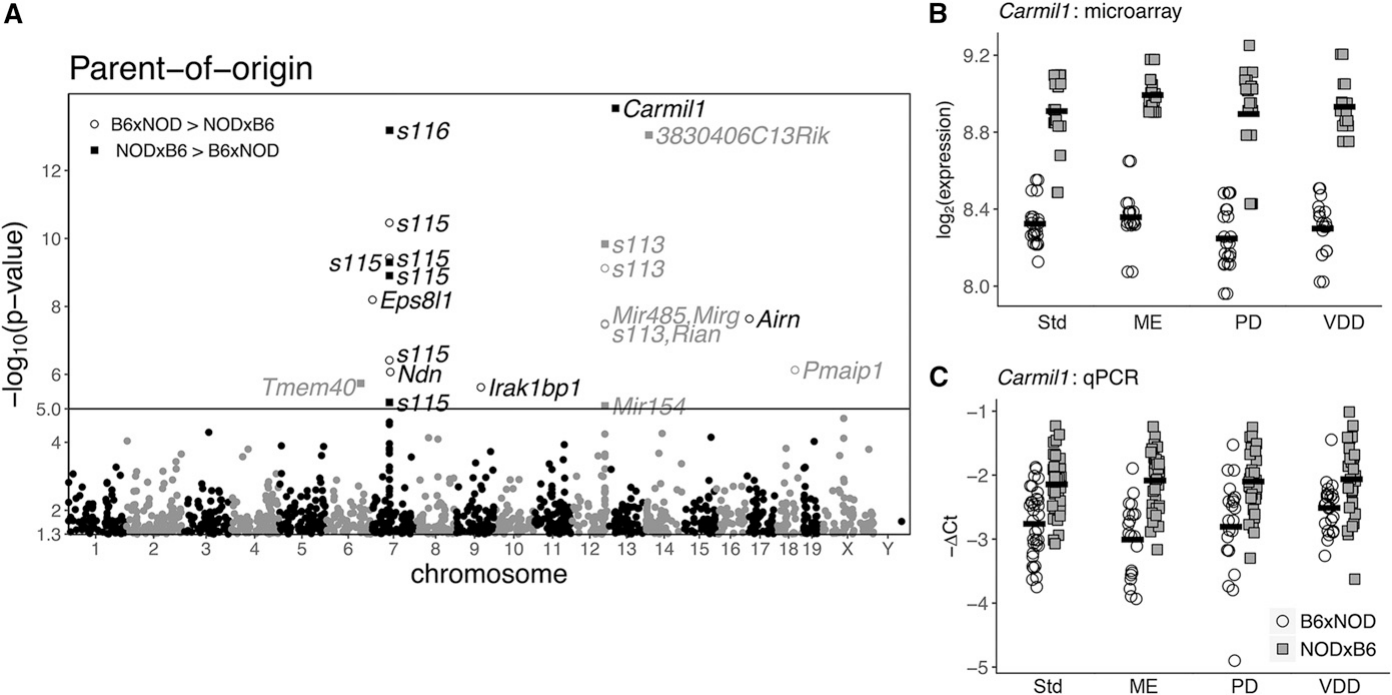
POE on *Carmil1* gene expression. (A) Manhattan-like plot of p-values of POE on microarray-based gene expression; each point corresponds to a probeset’s genomic location, coupled with the p-value of POE on expression at that location. Probesets with a nominal p-value >.05 are not shown. The solid line represents the FWER threshold. Probesets above the FWER threshold are labeled with the gene(s) that they interrogate. The *S113*, *S115*, and *S116* labels are shorthand for *Snord 113*, *Snord 115*, *Snord 116* respectively. Labeled points are shaped according to whether expression was greater in B6xNOD or NODxB6. The most significant POE is on *Carmil1*. (B) Raw microarray expression data for *Carmil1*; circles and squares represent expression for B6xNOD and NODxB6 hybrids, respectively. POE on expression is evident under all dietary exposures. (C) qPCR validation data for *Carmil1*, showing the same significant pattern of POE in all dietary exposures, confirming the microarray findings. In any qPCR assay, increased expression *reducesΔ*Ct; consequently, we use the y-axis to depict −ΔCt, ensuring that an increased y-value represents increased expression in both (B) and (C).

POE acted upon 15 genes (Table S9; Figure 6), a significant subset of which—nine—were imprinted (p$<2.2\times 10^{-16}$). Comparing these imprinted nine to the results from Crowley *et al.* (2015)—which employed reciprocal crosses between CAST/EiJ, WSB/EiJ, and PWK/PhJ—even though 48 imprinted genes were subject to POE in Crowley *et al.* (2015), only three imprinted genes were found to be subject to POE in both studies (*P* = 0.70).

Across all 15 genes, greater expression was not associated with either cross direction (seven were more expressed in NODxB6; ten were more expressed in B6xNOD). Both patterns were seen in the imprinted genes *Snord 113* and *Snord 115*, depending on the subregion (Table S9). Significant POE clustered on (Figure 6A) chromosome 7 in the vicinity of the imprinted *Snord 115/116* family, and on chromosome 12 near the imprinted *Snord 113* family.

#### POE on non-imprinted Carmil1 validated by qPCR:

The most significant POE was on *Carmil1* ($-\;{\text{log}}_{10}$(p) =13.8). This POE was consistent across diets (Figure 6B), and was validated by qPCR. qPCR was performed on 115 mice, 85 of which had already been assayed by microarray. POE on *Carmil1* was significant whether considering qPCR data from all 115 (*P* = 6.3e-7), only the 85 (*P* = 4.4e-07), or the qPCR-only 30 (*P* = 9.7e-11) (see Table S4).

#### Diet affects 37 (solely non-imprinted) genes:

The most significantly affected was *Cnot2* ($-\;{\text{log}}_{10}$(p) = 7.4). For 35 of the 37 genes (Table S10, Figure S6), significance was driven by the ME diet: across the 4 diets, these 35 genes were either most or least expressed in ME mice (See the “ME group rank” field in Table S10; Figure S6).

#### Diet-by-POE affects 16 genes, With only Mir341 imprinted:

Not only was *Mir341* the most significantly affected of all genes ($-\;{\text{log}}_{10}$(p) =6.5; Table S11) but it was also the only significantly affected imprinted gene in the microarray data. The set of 16 other genes subject to diet-by-POE, however, was not significantly enriched for *Mir341*’s predicted regulatory targets (*P* = 0.999, using miRHub; Baran-Gale *et al.* (2013)), a contradictory result.

**Figure 7:**
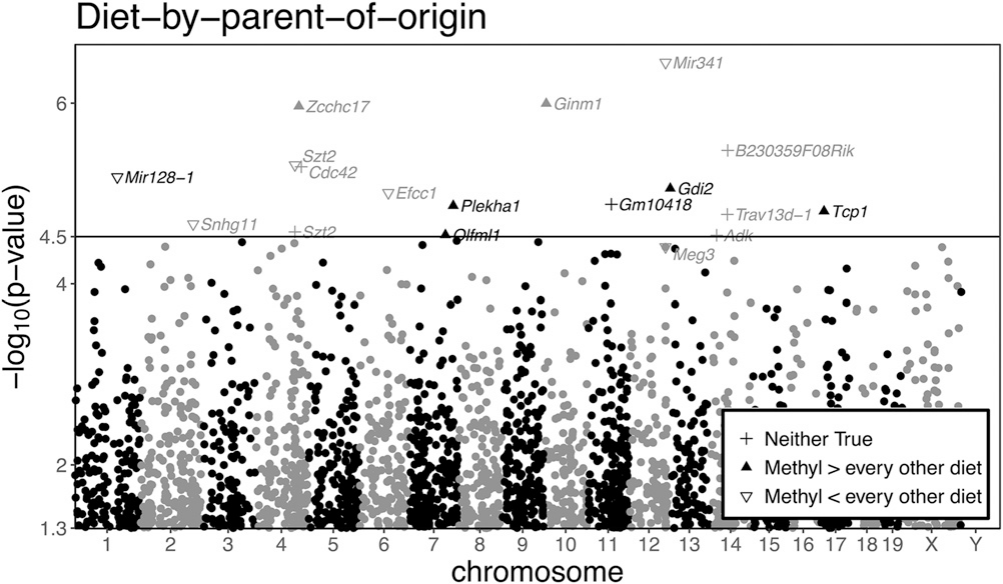
Manhattan-like plot of P-values of diet-by-POE effects on gene expression. Probesets above the FWER threshold are marked by a shape, which depends on whether ME exposed NOD mice have (on average) the highest expression relative to NOD mice on the other diets (up arrow), the lowest expression relative to NOD mice on the other diets (empty down arrow), or somewhere in middle of the 4 diets (plus sign). The solid line represents the FWER threshold. *Mir341* expression is the most significantly affected by diet-by-POE. Note that *Meg3*, an imprinted gene just below the FWER threshold, is also labeled.

Although no other imprinted genes were significantly affected, the imprinted gene *Meg3* came close to the FWER threshold (Figure 7; Figure S5A). However, this association was contradicted by qPCR, which showed no diet-by-POE on *Meg3* (Table S4).

In only 10 of the 16 genes subject to diet-by-POE was the methyl-by-POE effect the most or least extreme (binomial test two-sided p-value = 0.455); the ME diet did not significantly drive diet-by-POE significance.

### Mediation of POE by way of gene expression

#### POE on the gene expression of non-imprinted gene Carmil1 may be mediated by Airn:

The microarray and qPCR-based evidence for POE on *Carmil1* expression raised the question: given that *Carmil1* is not known to be imprinted, might *Carmil1* expression be regulated (*i.e.*, mediated) by some imprinted gene’s expression?

We first attempted to answer this question through a ChIPBase-driven analysis (Yang *et al.* 2013) of predicted and recorded transcription factor binding sites. We found that the protein product of *Wt1*, an imprinted gene, might bind upstream of *Carmil1*—suggesting that the POE on *Carmil1* might be mediated by *Wt1*. However, we deemed this hypothesis unlikely given that, in our data, *Wt1* expression levels were unaffected by POE (*P* = 0.267).

**Figure 8:**
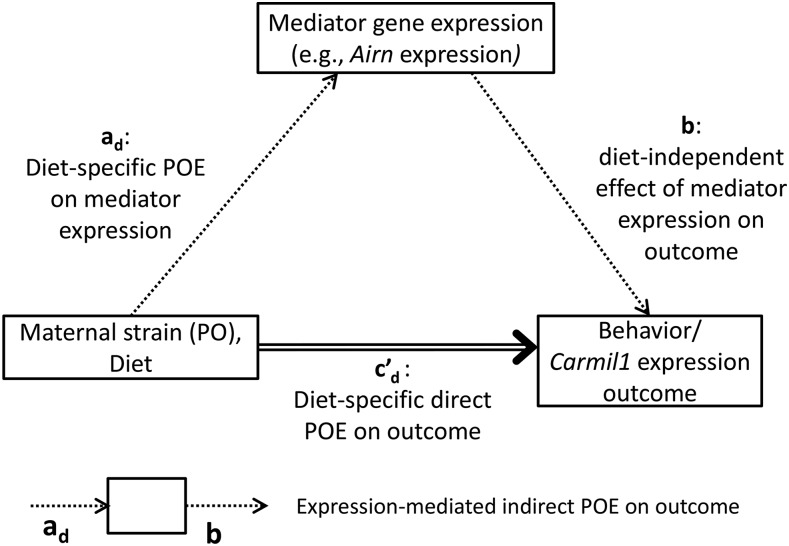
Model of gene-expression mediation of POE on the outcome, which is either behavior or *Carmil1* expression. Parent-of-origin, encoded as the maternal strain, in conjunction with diet, acts both directly upon the outcome, with effect size $c_{d}^{\prime },$ and indirectly upon the outcome, with effect size $a_{d}b.$ This indirect effect is composed of the diet specific POE on some mediator’s expression $\left(a_{d}\right)$ and the diet-independent effect of the mediator’s expression on the outcome (*b*). Not shown in this figure for clarity, but present in the actual model, are nuisance effects of dam, pipeline, breeding batch, and behavior specific covariates, that all can affect both mediator expression and the outcome. Mediation is determined by testing whether the average indirect effect $\left(ab=\bar{a_{d}}b\right)$ is significant according to its Minimum Tail Probability (MTP).

This focused bioinformatic analysis having failed to clearly identify a mediator, we applied a genome-wide analysis: for every microarray-measured gene, we tested whether its expression mediated the POE on *Carmil1* expression. The model used to test for mediation is shown in Figure 8.

**Figure 9:**
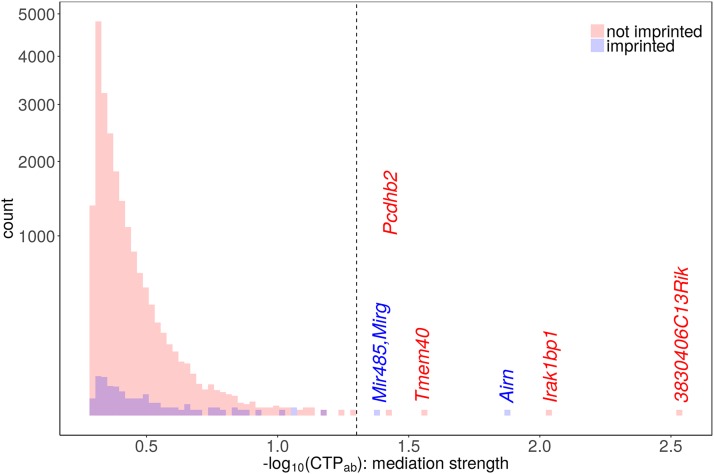
Histograms of the $-{\text{log}}_{10}$ Minimum Tail Probabilities (MTPs) for candidate gene mediators of POE on *Carmil1* expression. The red and blue histograms correspond to MTPs for non-imprinted and imprinted candidate mediators, respectively. Mediators whose mediation effect has a MTP < 0.05 (the dashed line threshold) are labeled. Notably, the imprinted gene *Airn* is one of the top 3 mediators of POE on *Carmil1*.

The expression of 8 different genes was found to significantly (Minimum Tail Probability, MTP < 0.05) mediate POE on *Carmil1* expression. For 7 of these 8 genes, their mediation (*i.e.*, indirect) effect acted against the direct effect (Figure 8); rather than explaining POE, expression of these 7 genes actually suppressed the overall POE on *Carmil1*. *3830406C13Rik*, a non-imprinted protein coding gene of unknown function (Yue *et al.* 2014), was the most significant (MTP = 0.00289) overall mediator of POE on *Carmil1*. *Airn* was the most significant (MTP = 0.0134) mediator that was imprinted; specifically, *Airn* acted to suppress POE on *Carmil1* (Table S6; Figure 9).

#### POE on behavior may be mediated by Carmil1 and/or Airn:

We repeated a similar genome-wide POE-mediation analysis for every behavioral outcome (including behaviors without significant POE in mediation-free analysis). A significant (MTP < 0.05) gene mediator of POE was observed for 10 of the 34 modeled behavioral outcomes. POE on some outcomes was mediated by more than one gene, and some genes mediated POE on more than one outcome. Although 16 different significant mediator-outcome pairs were observed, there were only 6 distinct genes/gene families significantly mediating any behavioral outcome: *Snord 113*, *Snord 115*, *Snord 116*, *3830406C13Rik*, *Rian*, *Carmil1*. In 15 of the 16 significant mediator-outcome pairings, the gene expression mediator suppressed POE; *i.e.*, 15 of 16 gene mediators acted in the opposite direction of the direct POE on behavior (Table S7).

To determine each gene’s mediation of POE on behavior in the aggregate, we combined the MTPs for a given gene, over all behaviors, into a single metric: the “Combined Tail Probability” (CTP; Appendix J). By this combined metric, 21 probesets—corresponding to 17 distinct genes/gene families—mediated POE on behavior in the aggregate (CTP < 0.05). Even though *Airn* was not a significant mediator for any individual behavior (see above), it was the most significant mediator in the aggregate (CTP = 5.09e-05). *Airn* was followed closely by (a subregion of) *Snord 115* (CTP = 0.000408) and *Carmil1* (CTP = 0.000518). See Table S8.

To gain further insight into aggregate mediation, for each outcome we determined the 3 most significant POE gene-mediators. Each gene was then scored according to the number of behaviors for which it was one of the 3 top mediators. According to this metric, *Airn* was the most notable mediator, acting as one of the 3 most-significant POE-mediators for 12 behavioral outcomes, while *Carmil1* was a top-3 mediator for 8 outcomes. The enrichment for *Airn*, *Carmil1*, and *Snord 115* in the sets of top-3 mediators is also readily apparent in Figure S7: for each behavior, genes with a significant MTP are labeled, as are *Carmil1* and *Airn* if they were among the top 3 mediators; mediation MTPs for *Airn* and *Carmil1* are often extreme.

## Discussion

Our study identifies POE on behavior, and, in the same genetic background, on gene expression. These two are naturally linked: POE on behavior (or any complex trait) must ultimately reflect POE on gene expression. Significantly, we found only a modest number of POE in either (5 and 15, respectively), and most of these, with notable exceptions, were robust to differences in perinatal diet. With numbers this small, we believe that it should be possible to map POE on behavior to locations in the genome by comparing POE incidence across a relatively small number of genetically distinct reciprocal crosses—an approach we have been investigating in a different ongoing study (Schoenrock *et al.* 2018) that examines behavior and expression data from RF1s of 9 different pairs of mouse strains.

Beyond its specific results, our study serves to advance a general protocol based on RF1s for studying POE and perinatal environment effects on any complex trait. The RF1 allows us to hold genetic background constant as we study the architecture of POE. To investigate the interaction of developmental-environment with POE, we further varied perinatal nutrition using four different diets, where diet was a relatively easy variable to control and there is ample evidence to suggest its importance in POE. By repeating the behavioral and expression assays under multiple dietary conditions, we: 1) enabled potential detection of environment-by-POE; 2) hedged our bets, as an effect that would be unobservable in one environment might be amplified in another; and 3) enabled potential detection of POE that would generalize across environments.

In the remainder, we discuss the range of mechanisms that might explain POE as discoverable by our approach and go into more detail regarding the implications of specific results for POE, diet, and diet-by-POE.

### POE observability: parental-POE, and imprinting-based coding-POE and eQTL-POE

The two examined groups of female RF1s, NODxB6 and B6xNOD, were (aside from mitochondria) genetically identical. Consequently, differences in phenotype between these two groups could with high probability be attributed to POE. The design of our study did not, however, allow us to determine the specific mechanism underlying any POE—namely, whether it is a maternal or paternal factor, or imprinting. This could be potentially determined in future studies that employ specific interventions of the sort described earlier, such as cross-fostering, embryonic transfer, etc. But even without such interventions, we can still speculate as to the possible ways POE could (and could not) have arisen through parental or imprinting effects.

It is relatively straightforward to see how POE could arise through parental effects (“parental-POE”). For example, if there were a difference between NOD and B6 at some locus affecting maternal care, RF1 pups raised by B6 *vs.* NOD mothers might differ in their expression profile (Figure S1), which could lead to differences in behavior between RF1 directions.

**Figure 10:**
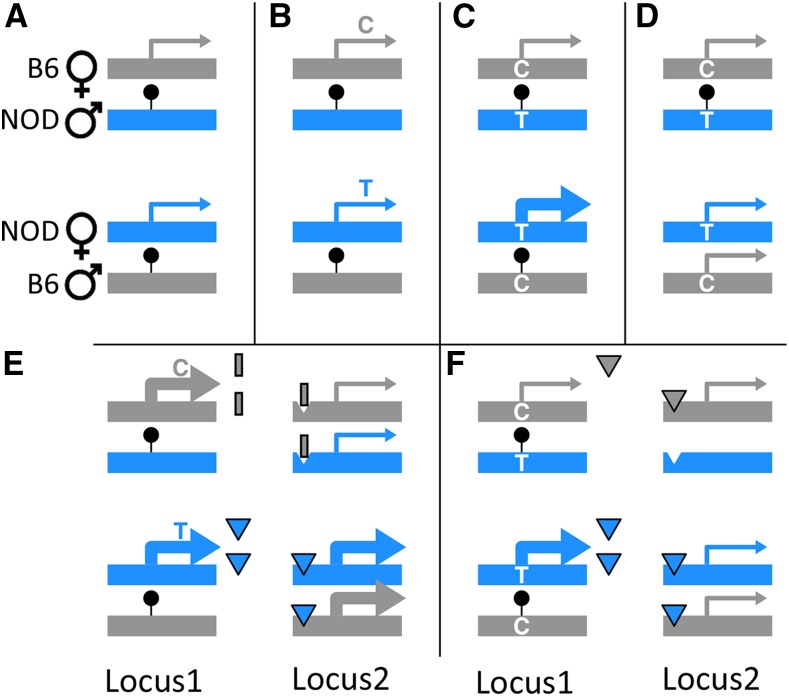
Examples in RF1s of imprinting-based-POE: cis/trans coding-POE and eQTL-POE. Examples depict an imprinted gene which is fully active when maternally inherited, but fully silenced when paternally inherited. (A) A lack of observable POE in spite of imprinting: B6 and NOD are identical in sequence, so whichever allele is silenced, the resulting expression product is the same in both RF1 directions. (B) Coding-POE: B6 and NOD differ in coding sequence causing allele-specific expression differences between RF1 directions (unobservable by microarray). (C) eQTL POE: the NOD promoter attracts a more effective transcription factor (TF), so NODxB6, in in which the NOD allele is expressed, yields more expression. (D) eQTL-POE driven by background-dependent imprinting: imprinting is lost in B6, so NODxB6, in which imprint-silencing affects neither allele, yields more expression. (E) Trans coding-POE upon locus 2: locus 2 is identical between NOD and B6, but it is regulated by an imprinted TF whose NOD version binds more tightly; so in NODxB6, in which the NOD TF is expressed, locus 2 expression is increased. (F) Trans eQTL-POE upon locus 2: locus 2 is regulated by an imprinted TF whose NOD version has a stronger promoter; so in NODxB6, in which the NOD allele for the TF is expressed, increased availability of the TF increases microarray-observable locus 2 expression.

Our observed POE could also have been driven by imprinting. Akin to parental-POE, for imprinting-based POE to be observable, imprinting would need to interact with a locus differing in sequence between parents (Figure 10A). This difference driving imprinting-based POE could have been in: 1) an imprinted gene coding region, making the POE a “coding-POE”; or 2) in a imprinted gene regulatory region, making the POE an “eQTL-POE”.

In coding-POE, the expressed allele’s coding sequence differs between the two cross directions. Consequently, the RF1 populations are equal in total expression but allele-specific expression (ASE) differs (Figure 10B). Although ASE could not be quantified directly by the microarrays in our study, ASE differences could still manifest as an observable POE on an emergent phenotype such as behavior, or as POE on total expression of a downstream gene.

By contrast, eQTL-POE could arise by way of non-coding *cis*-eQTLs that alter total expression of an imprinted gene, or by maternal *vs.* paternal effects. For example, a eQTL-POE could arise from differences in promoter attractiveness (Figure 10C). Alternatively, eQTL-POE could arise by way of (total or partial) genetic background-dependent loss of imprinting (Vrana 2007; Duselis *et al.* 2005; Wolf *et al.* 2014) (Figure 10D), perhaps due to histone modifications (Weaver and Bartolomei 2014). In our study, all directly-observed POE on expression are necessarily instances of eQTL-POE, because we did not employ assays capable of measuring ASE.

eQTL-POE and coding-POE both require a genetic difference between parents in some imprinted gene. However, any gene can exhibit POE—provided it is regulated by an imprinted gene. This trans effect can occur either by way of coding-POE (Figure 10E) or eQTL-POE (Figure 10F).

Although we did observe POE on both behavior and expression, it is quite possible that numerous POE instances were undetectable by our study. Among the reasons: as mentioned above, by using microarrays, we could not observe coding-POE; by measuring expression only once, ∼8 weeks after birth, we may have failed to observe any transient POE from developmental-stage-specific imprinting or maternal care; and by measuring whole-brain gene expression, we may have failed to observe POE that act in opposite directions, depending on the brain subregion—for example, some POE arise from imprinting that is brain subregion-specific (Koerner *et al.* 2009; Prickett and Oakey 2012).

### POE on expression

The set of genes subject to POE was significantly enriched for genes we believe to be imprinted. This result is expected: POE, as we define it, is observable only when gene expression depends on both genetic background and parent-of-origin; and known imprinted genes satisfy the latter criterion automatically. Regarding dependence on genetic-background, however, an enrichment of imprinted genes may be surprising. Given that only a single copy of imprinted genes is fully expressed, regulation of imprinted gene expression should be under tight control, evolutionarily speaking, and mutations affecting expression levels of imprinted genes should be rare. Moreover, between the closely related classical inbred strains of our study, B6 and NOD [as opposed to between CAST/EiJ, PWK/PhJ, and WSB/EiJ from (Crowley *et al.* 2015)], there has been relatively little time for genetic differences to accumulate.

We note that all 9 imprinted genes that were subject to POE in our study contain non-coding variants that differ between NOD and B6, a finding consistent with *cis*-acting, eQTL-POE (Figure 10C,D) (although nothing precludes parental-POE from acting on these imprinted genes as well).

There were also 6 non-imprinted genes subject to POE, notably including *Carmil1*, our top POE hit. The simplest explanation for these POE may be parental-POE. But to explore whether the data were also consistent with *trans*-acting eQTL-POE (as in Figure 10F), we employed mediation analysis, identifying potential imprinted mediators of *Carmil1*.

#### Mediation of POE on Carmil1:

To determine potential imprinted regulators of *Carmil1* (as in Figure 10F), we applied mediation analysis, identifying *Airn*. Unexpectedly however, *Airn* exerted its mediation effect in the opposite direction of the overall POE on *Carmil1* (ab and $c^{\prime }$ have opposite signs in Table S6), suggesting that *Airn* acts *in trans* to suppress POE on *Carmil1*. All but one of the other genes found to be significant mediators also acted to suppress the overall POE. The lack of explanatory mediation in the same direction as the overall POE may be due to the many unobservable forms of POE on expression: genes that fail to reveal POE in their own expression cannot be statistically significant mediators of POE on another gene’s expression. Alternatively, the overall POE may be primarily driven by parental-POE, which is suppressed by *Airn* together with the other imprinted and significant mediators.

### POE on behavior and its mediation by gene expression

Five behaviors were significantly or suggestively affected by POE, four of which were locomotor behaviors. The enrichment for POE among locomotor behaviors could in part be due to increased power: locomotor activity has been found to be among the most stable of behaviors across laboratories and time (Crabbe *et al.* 1999; Wahlsten *et al.* 2006), resulting in more power to observe group differences. Alternatively, locomotion may be a particularly good surrogate measure of rodent emotionality (Hall 1934). Interestingly, offspring locomotion seems to mimic that of the maternal strain, suggesting locomotion is affected either by maternal factors or by maternally-expressed imprinted genes.

But for *all* POE significant behaviors (locomotor or not), behavioral POE must have been driven by some gene subject to POE. Our mediation analysis implicated 17 genes as potentially being mediators of behavioral POE. For 16 of the 17 genes, however, the estimated mediation effect was to suppress POE, meaning that those genes did not explain the overall POE on behavior. We posit that an explanatory POE on gene expression may simply have been unobservable, for the reasons described earlier.

### Airn and Carmil1 as mediators of POE

The most commonly shared mediators of behavior were *Carmil1* and *Airn*, with *Airn* also being the top mediator of POE on *Carmil1*.

*Airn*’s mediation of POE is likely *trans*-acting. *Airn* is an imprinted, paternally-expressed, long non-coding RNA (lncRNA), which to our knowledge has not been found to affect any complex trait directly. Rather, *Airn* is known to control imprinting of three nearby maternally-expressed genes: *Slc22a2*, *Slc22a3*, and *Igf2r* (Cleaton *et al.* 2014). But none of the three genes were at all significant mediators of POE on any outcome of interest in our dataset. So, akin to other lncRNAs and imprinted genes found to affect distal gene expression (Vance and Ponting 2014; Gabory *et al.* 2009), we posit that *Airn* may be exerting POE on behavior by affecting distal genes, such as *Carmil1* or *Snord 115* (as in Figure 10E). Our study is underpowered to directly examine this two-step mediation hypothesis.

*Carmil1* may provide a link between cytoskeleton dynamics and cell migration, and behavioral change. *Carmil1* has a known cellular role in: 1) interacting with Capping Protein, which regulates actin elongation; and 2) activating the small GTPase *Rac1*, an important regulator of cytoskeletal dynamics (Gonzalez-Billault *et al.* 2012). Such actin cytoskeleton dynamics, critical for cytokinesis and cell migration (Rottner *et al.* 2017), are important throughout the lifespan for neurodevelopment and neural plasticity (Menon and Gupton 2016; Gordon-Weeks and Fournier 2014). In *C. elegans*, neuronal cell and axon growth cone migration has been shown to be negatively regulated by *CRML-1*, the homolog of *Carmil1* (Vanderzalm *et al.* 2009). Our study, in a mammal, is the first to find a direct association between variation in *Carmil1* expression and behavior.

### Caveats to mediation analysis of POE on Carmil1 and behavior

We note that our analysis was applied one candidate mediator at a time; thus, any significant mediators may simply be co-expressed with the true mediator gene(s). We also note that for both mediation analyses (*Carmil1*/behavior outcome) we assumed a direction of causality in which some imprinted gene mediates POE on the outcome; although this might seem intuitive, it cannot be verified, and the “outcome” might actually mediate the imprinted gene.

Our directionality assumption is especially uncertain in the behavioral analysis: expression in the brain was, out of necessity, measured after behavior; consequently, stressful behavioral assays could have altered expression. In future studies, we intend to address this weakness by a matching-based imputation: behavior-unperturbed expression will be imputed in behaviorally-assayed mice using expression data from mice that were unexposed but are genetically identical and otherwise perfectly matched (*cf*. a related matching-based design in Crowley *et al.* 2014).

### Diet effects

Our data revealed numerous significant diet effects on gene expression, with significance primarily driven by very high (or low) expression under the ME diet. Although the ME diet’s composition did differ slightly from that of the other diets in ways other than methyl content, given the direct role of methyl donors on DNA methylation and, by extension, on the regulation of gene expression, it seems likely that the diet effects observed in our study are truly driven by differences in methyl concentration. The specific genes affected by perinatal methyl enrichment may be important, given that in the U.S. and other countries, fortification of enriched cereal grain by folic acid (a methyl donor) is mandatory (Crider *et al.* 2011). Surprisingly, none of the genes subject to diet effects were imprinted; this may be due to transient or tissue-specific imprinting (Koerner *et al.* 2009; Prickett and Oakey 2012; Andergassen *et al.* 2017), or perhaps DNA methylation at imprinted sites is in fact more stable than at non-imprinted sites.

Overall, diet significantly altered the expression of 37 genes. Yet the only complex phenotypes affected were body weight and PPI, and those effects were only suggestive. The lack of significant diet effects on behavior, even in the presence of expression changes, is surprising but not inexplicable. Among other possibilities, the diet effects on behavior may be too small to overcome a sample size that was split among four different diets. Alternatively, the relatively limited set of behaviors we measured may simply not have included diet-sensitive behaviors. Or, perhaps the diets we used were insufficiently extreme to elicit a change in an emergent trait such as behavior.

### Diet-by-parent-of-origin effects

Given that our experiment perturbed nutrients involved in imprinting, we had expected to find multiple diet-by-POE on imprinted genes. However, only 1 of the 16 genes subject to diet-by POE was imprinted. This single gene was *Mir341*, with the next most significant imprinted gene (but not passing FWER) being *Meg3*. But even these sparse imprinted gene results were contradicted by our other data: *Mir341*’s predicted targets of regulation were unaffected by diet-by-POE, and *Meg3*’s weak diet-by-POE in microarray data failed to replicate at all in qPCR data (Figure S5).

The dearth of observed diet-by-POE on imprinted genes may be an inevitable consequence of lower power for testing these interaction effects, with specific combinations representing smaller sample sizes. Or, perhaps it is due to the aforementioned factors that might have concealed imprinted-gene *diet* effects; *i.e.*, transience and/or tissue-specificity of imprinting. And as was the case for diet effects on behavior, insufficiently extreme diets may have caused the lack of diet-by-POE on our behaviors (save for a suggestive effect on percent center time).

For the 15 non-imprinted genes subject to diet-by-POE, effect significance did not seem to be primarily driven by the ME diet. This is in striking contrast to the centrality of methyl enrichment on diet effects on imprinting. Given this finding, along with the dearth of observed diet and diet-by-POE on imprinted genes, the diet-by-POE on non-imprinted genes is less likely due to regulation by imprinted genes (Figure 10E,F) and more likely due to diet-modulated maternal factors.

### Conclusion

In summary, our study has demonstrated a reciprocal hybrid strategy for studying POE and diet-by-POE on expression and on behavior. Our results suggest the feasibility of extending such an approach to one that explicitly maps POE and diet-by-POE by repeating the RF1 design across multiple genetic backgrounds.

## Figures and Tables

**Figure A1:**
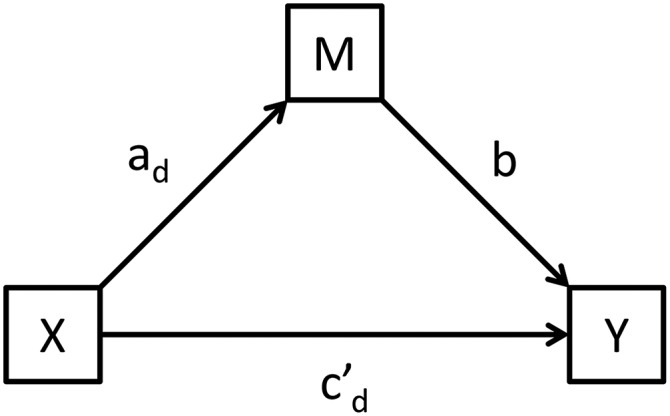
Multilevel mediation model in which the levels are diets. *X* represents the maternal-strain treatment, *Y* is the outcome (behavior or expression), and *M* is the mediating gene expression factor. $a_{d}$ is the (diet-specific) effect of the treatment on the mediator, $c_{d}^{\prime }$ is the (diet-specific effect) direct effect of the treatment on the outcome, and *b* is the (diet-independent) effect of the mediator value on the outcome.

## APPENDIX A

### Behavior Models

The LMM used to model RF1 behavioral phenotypes (excluding the startle/PPI phenotypes) was as follows. The behavioral outcome $y_{mi}$ of mouse mi was modeled as:

$$f\left(y_{mi}\right)={\mathrm{intcov}}_{mi}+{\text{diet}}_{d\left[m\right]}+{\text{POE}}_{s\left[m\right]}+\text{diet}.\text{by}.{\text{POE}}_{\left(sd\right)\left[m\right]}+{\text{dam}}_{m}+\varepsilon_{mi},$$ (1)

where mi denotes the *i*th mouse of mother *m*; d[m] denotes mother *m*’s diet, where d=1,…,4, corresponding to diets Std, ME, VDD and PD; s[m] denotes the mother’s strain, where s=1,2 corresponds to B6 and NOD respectively; (sd)[m] denotes the mother’s diet and strain combination. Modeled effects consisted of: $\mathrm{int}{\mathrm{cov}}_{mi},$ a fixed intercept and a set of (behavior-specific) fixed effect covariates; ${\text{diet}}_{d},$ a fixed effect of diet *d*; ${\text{POE}}_{s},$ a fixed effect of POE (technically, strain-by-POE); $\text{diet}.\text{by}.{\text{POE}}_{sd}$ a fixed effect of diet-by-POE; and ${\text{dam}}_{m},$ a random effect of dam. The function f() is a transformation chosen to ensure the residuals $\varepsilon_{mi}$ are approximately normal (Appendix B).

#### Startle/PPI Models

For every prepulse intensity, 6 measurements of the average startle response were taken per mouse (all in the same chamber). The startle/PPI LMMs therefore accounted for repeated measures. Letting $y_{mi,j}$ be mouse mi’s *j*th measurement, we modeled:

$$f\left(y_{mi,j}\right)={\mathrm{intcov}}_{mi}+{\text{diet}}_{d[m]}+{\text{POE}}_{s\left[m\right]}+\text{diet}.\text{by}.{\text{POE}}_{b\left[m\right]}+{\text{chamber}}_{h\left[mi\right]}+{\text{dam}}_{m}+{\text{pup}}_{mi}+\varepsilon_{mi,j},$$ (2)

where ${\text{chamber}}_{h[mi]}$ is a random effect of chamber, and ${\text{pup}}_{mi}$ is the random effect of mouse mi.

## APPENDIX B

### Variable Transformation Procedure

A transformation procedure was applied to both the expression and the behavior phenotypes to ensure residual normality. For a given LMM requiring a transformation of the outcome *y*, the procedure was as follows. Center and scale *y* to mean 0 and standard deviation 1 to give *z*. Apply a shifted Box-Cox transformation (Sakia, 1992; Box and Cox, 1964), restricted to the ladder of powers λ∈{−3,−2,−1,−.5,0,.5,1,2,3} to give in each case values $z^{\left(\lambda \right)}.$ For each transformation $z^{\left(\lambda \right)},$ the LMM is fitted, and residual normality is evaluated using the Shapiro-Wilk *W* statistic (Shapiro and Wilk, 1965); denote the optimal *λ* as $\hat{\lambda }.$ If $\hat{\lambda }\in \left\{0,.5,1,2\right\},$ then use $z^{\left(\hat{\lambda }\right)};$ if $\hat{\lambda }\in \left\{-2,-1,-.5\right\},$ then additionally negate the value, in order to ensure the monotonicity of the transformation and thereby improve interpretability of effect estimates; if the $\hat{\lambda }\in \left\{-3,3\right\},$ then discard the transformation and instead apply a rank inverse normal transform (Van der Waerden, 1952). Rescale the selected transformed variable to mean 0 and standard deviation 1.

Transformed versions of behavior and expression data are provided in File S10 and File S11.

## APPENDIX C

### Microarray Expression Models

Expression was first adjusted by regressing out nuisance factors, and then the *adjuste*d expression was modeled to test diet, POE, and diet-by-POE. This two-step process was employed to facilitate permutation testing later on.

#### Generation of the adjusted expression outcome

Letting $y_{mi,j}$ be the average expression of probes in probeset *j* for mouse mi,we obtained adjusted expression values as residuals ${\hat{\varepsilon }}_{mi,j}$ from the linear model:

$$f\left(y_{mi,j}\right)=\mathrm{int}{\mathrm{cov}}_{mi,j}+{\text{SV}}_{mi,j}+\varepsilon_{mi,j},$$ (3)

where the covariates in $\mathrm{int}{\mathrm{cov}}_{mi,j}$ were the nuisance effects of pipeline and breeding batch. The ${\text{SV}}_{mi,j}$ term modeled fixed effects for 7 “surrogate variables” (SVs), which represented aggregate effects of unobserved confounding on the microarray (Appendix F). Specifically, ${\text{SV}}_{mi,j}=\sum_{k=1}^{7}\beta_{k,j}v_{mi,k},$ where $v_{mi,k}$ is mouse mi’s value for the *k*th SV, and $\beta_{k,j}$ is the fixed effect of that SV on the expression of probeset *j*. (Estimation of the SVs themselves is described in Appendix D.)

#### Model of adjusted expression outcome

For each probeset *j*, adjusted expression (a.k.a., the residuals from Eq 3) was then analyzed using the LMM,

$$f^{\left(\varepsilon \right)}\left({\hat{\varepsilon }}_{mi,j}\right)=\mu_{j}+{\text{diet}}_{d\left[m\right],j}+{\text{POE}}_{s\left[m\right],j}+\text{diet}.\text{by}.{\text{POE}}_{\left(sd\right)\left[m\right],j}+{\text{dam}}_{m,j}+\epsilon_{mi,j},$$ (4)

where $\mu_{j}$ and $\epsilon_{mi,j}$ are the intercept and residual error, $f^{\left(\varepsilon \right)}$ is a transformation that may be different from *f* in Eq 3, and other terms are defined as in Eq 1.

## APPENDIX D

### Surrogate Variable Estimation Allowing For Random Effects

Gene expression measurements by microarray are typically affected by many unobserved factors, some of which can have a large confounding effect on transcript levels across many genes. One way to control for such unobserved factors is to first model their aggregate effects as linear combinations of “surrogate variables” (SVs; Leek and Storey 2007), and then include these SVs as predictors in subsequent modeling, and/or regress these effects out (as in Appendix C).

Here we mostly— deviating somewhat to accommodate random effects and variable transformation— follow the Supervised Surrogate Variable Analysis (SSVA) approach of Leek (2014), which defines the SVs using negative control probes; success of this approach requires that unobserved confounding effects arise from technical rather than biological variation. As a further aside, we note that our approach is also largely equivalent to the “remove unwanted variation with negative control genes” (RUVg) strategy (Risso *et al.*, 2014), applied to microarray data.

In our implementation of SSVA, we first estimate a standardized matrix of the aggregate effects that arise from unobserved factors, *E*. For each negative control probe c=1,…,C, we fitted the LMM:

$$f\left(y_{mi,c}\right)={\mathrm{intcov}}_{mi,c}+{\text{diet}}_{d\left[m\right],c}+{\text{POE}}_{s\left[m\right],c}+\text{diet}.\text{by}.{\text{POE}}_{\left(sd\right)\left[m\right],c}+{\text{dam}}_{m,c}+\varepsilon_{mi,c},$$

where terms are defined as in Eq 1 and Eq 4, and where the estimated residuals, ${\hat{\varepsilon }}_{mi,c},$ were standardized and stored in *n*-vector $e_{c}.$ These steps were repeated for all *C* negative control probes to give the n×C matrix E.

Let the SVD of E be denoted as $U\Sigma V^{\prime }.$ Under this parameterization, the space of aggregate unobserved factor effects on the control probes is (by construction) spanned by the *n* columns of U. Since a model for main probes that included all *n* columns as surrogate variables would be unidentifiable, the first K=7 columns of **U** were chosen as an approximating subset of surrogate variables. K=7 was chosen using the strategy described in Sun *et al.* (2012): a plot of the squared eigenvalues from Σ was examined, and it revealed an inflection point at 7 eigenvalues.

The original implementation of SSVA did not regress any effects out of control probes, under the assumption that these probes should be unaffected; in contrast, we regress these effects out before computing eigenvectors. We justify this by noting that if in fact the treatments of interest somehow did affect the control probes, we would not want these treatment effects to be incorporated into the surrogate variables. And if the control probes truly are unaffected by any of the observed experimental factors, then there should be no harm in residualizing out these size-zero effects.

## APPENDIX E

### Bias-Adjustment For Gene Expression P-Values

For some effect types that were tested in the gene expression model of Eq 4, the distribution of nominal p-values across all transcripts was consistent with those p-values being downwardly biased. To mitigate this bias we applied an empirical adjustment similar to the genomic control procedure of Devlin *et al.* (2001) (see also Dadd *et al.* 2009). Let $p_{j}$ be the p-value associated with a given effect type (diet, POE, or diet-by-POE) on the *j*th probeset, let F(x) be the cumulative distribution function for the $\chi_{1}^{2}$ density, and define $x_{j}=F^{-1}\left(p_{j}\right)$ and $x=\left(x_{1},\ldots ,x_{m}\right).$ Under unbiasedness, p-values associated with testing for given effect should, under the null, have a uniform distribution, $p_{j}∼\text{Unif}\left(0,1\right),$ such that $x_{j}∼\chi_{1}^{2}.$ Assuming most results are in fact null, in the dataset as a whole we would expect $\text{median}\left(x\right)≃F^{-1}\left(0.5\right).$ However, if significances were systematically inflated, the null $x_{j}^{\prime }$s would appear as if from a scaled $\chi_{1}^{2}$ such that $x_{j}/\lambda ∼\chi_{1}^{2}$ with inflation factor λ>1. Therefore, we correct for this systematic inflation by first estimating the inflation factor as $\hat{\lambda }=\text{median}\left(x\right)/F^{-1}\left(0.5\right)$ and then calculating bias-adjusted p-values as ${\tilde{p}}_{j}=F\left(x_{j}/\hat{\lambda }\right).$

## APPENDIX F

### Permutation-Based Fwer Thresholds For Gene Expression P-Values

For gene expression, empirical p-value thresholds that controlled for the family-wise error rate (FWER) across all probesets were determined by permutation. A separate FWER threshold was computed per effect of interest (diet, parent-of-origin, and diet-by-parent-of-origin). Below, we describe the permutations that were generated, the statistic that was collected per permutation, and how this was translated into a significance threshold.

#### Structure of permutation

For every permutation-tested effect type, we generated a separate set of W=401 permutations (including the identity permutation), w=1…,W. Litters were taken as exchangeable units; diet/strain labels were permuted amongst the dams, and all pups of a given dam were assigned their dam’s diet/strain label. Permuting *labels*, rather than outcomes, enabled us to allow for varying litter sizes between dams.

For the main effects we employed a form of restricted permutation (Anderson and Braak, 2003; Good, 2005); *i.e.*, for parent-of-origin effects, we randomly permuted the strain labels (s in Eq 4) between dams that had been *exposed to the same diet*, whereas for diet effects, we randomly permuted diet labels (d) between dams *of the same strain*.

For the interaction effect of diet-by-POE, we employed a form of unrestricted permutation (Anderson and Braak, 2003; Good, 2005) of the interaction labels. In particular, we permuted the interaction labels sd between dams but while holding the s and d labels of strain and diet constant.

#### Permutation statistic and threshold computation

For each permutation *w* and probeset j=1,…,J we fitted the expression LMM of Eq 4. Note that the modeled outcome in this equation is adjusted gene expression from which *all nuisance covariates have already been regressed*; following Gail *et al.* (1988), this residualization was performed to facilitate exchangeability for the effects of interest. For every permutation, the fitting of Equation 4 included recalculation of the transformation $f^{\left(\varepsilon \right)}.$ Furthermore, for every permutation, we bias-adjusted (through genomic control, Appendix E) the p-values, $\tilde{p}={\tilde{p}}_{1}^{\left(w\right)},\ldots ,{\tilde{p}}_{J}^{\left(w\right)}$ and recorded the minimum, $p_{\min}^{\left(w\right)}.$

The set of *W* such minimum p-values from all permutations was then used to estimate the FWER α=0.05 threshold via modeling of a generalized extreme value (GEV) distribution after Dudbridge and Koeleman (2004); Manly (2006). Specifically, a GEV was fitted to $T_{w}=-\log\left[p_{\min}^{\left(w\right)}\right]$ for w=1,…,W using R package evir (Pfaff and McNeil, 2012), and the fitted GEV was used to estimate the upper 5% quantile, $T_{\alpha =.05}.$ $T_{\alpha =.05}$ was then translated back into a threshold on the p-value scale as $p_{\alpha =.05}=e^{-T_{\alpha =.05}}.$ Note that, as a conservative measure, the GEV fit included the identity permutation.

## APPENDIX G

### Probe Alignments and Estimated Probeset Positions

Probe alignments were downloaded from the Ensembl 38.75 funcgen database (Yates *et al.*, 2016). Notably, this database contained alignments for MoGene1.0 ST probes, rather than for the MoGene1.1 ST probes that we used in our experiment. To address this mismatch, we imputed 1.1 alignments by using the fact that every 1.1 probe is identical to at least one 1.0 probe in sequence (though not in probe id); we formed correspondences from each 1.1 probe to its identical-sequence 1.0 probe alignment. Since most probes aligned to multiple positions, we estimated per probe and per probeset, the “intended” target position, defining this self-referentially as the position that minimizes the sum of distances between probes in the same probeset.

## APPENDIX H

### Criteria For Masking Biased And Uninformative Probes/Probesets

APT masking was used to eliminate four types of probes: 1) probes aligning to ≥100 locations; 2) probes aligning outside of annotated exons; 3) probes whose “interior” (basepairs 3-21) aligned to regions in which NOD possesses a variant relative to B6, *i.e.*, probes with a binding affinity difference between strains (Dannemann *et al.*, 2009), where NOD variants were extracted from the Inbred Strain Variant Database (Oreper *et al.*, 2017); or 4) redundant probes mapping to the same position. Following probe masking, probe*sets* were eliminated if they contained <4 non-masked probes, or if every remaining non-masked probe measured <32 units of expression across all samples.

## APPENDIX I

### QPCR ANALYSIS

#### qPCR model

Letting $y_{mi,j}^{\prime }$ be the qPCR relative cycle threshold for a targetted gene (*Meg3* or *Carmil1*), we modeled:

$$f\left(y_{mi,j}^{\prime }\right)={\mathrm{intcov}}_{mi}+{\text{diet}}_{d\left[m\right]}+{\text{POE}}_{s\left[m\right]}+\text{diet}.\text{by}.{\text{POE}}_{b\left[m\right]}+{\text{dam}}_{m}+\text{batch}.{\text{plate}}_{a\left[mi\right]}+\varepsilon_{mi,j},$$

where intcov includes the intercept and behavioral pipeline, $\text{batch}.{\text{plate}}_{a}$ is a random effect of the combination *a* of breeding batch and qPCR plate, and the other terms are akin those defined in the microarray model (Appendix C).

#### qPCR normalization

The raw value measured by qPCR is a target gene’s cycle threshold. To allow comparison between qPCR batches, which can vary in replication efficiency, the cycle threshold for a target gene must be normalized by some reference gene that is unaffected by biological factors. As such, rather than modeling the cycle threshold, we model the relative cycle threshold, defined as $\Delta Ct=Ct_{\text{target}}-Ct_{\text{reference}}.$ The ΔCt relative cycle threshold represents the relative gene expression level of the target gene on the log scale (Didion *et al.*, 2015). The larger ΔCt is, the less the target gene expression.

We chose *Rfng* as the reference gene, because microarray data suggested negligible effects of diet, POE and diet-by-POE on *Rfng* expression. Specifically, each candidate reference gene was assigned a score equal to the minimum of the p-values for POE, diet-by-POE, and diet effects on the candidate reference’s microarray-measured expression. *Rfng* had the largest such score.

## APPENDIX J

### Bayesian Mediation Model

Mediation analysis is typically posed as the estimation of the model in Figure A1: An intervention or predictor variable *X* affects an outcome *Y* either directly or/and through an observed mediator outcome *M* (Hayes, 2009). In our case, *X* is reciprocal direction (*i.e.*, parent-of-origin, coded as the maternal strain), *M* is the expression of a mediator gene, and *Y* is the outcome of primary interest, either expression of *Carmil1* or a behavioral phenotype. By common convention, the effect of *X* on *M*, *i.e.*, the POE on *M*, is denoted *a*, which in our case is $a_{d}$ to allow different effects under each diet *d*, and the effect of *M* on *Y* is denoted *b*. The product $a_{d}b$ is then the expression-mediated effect of parent-of-origin on *Y*, conditional on the diet *d*, and our primary quantity of interest is this value averaged over diets, $ab=\bar{a_{d}}b.$ The direct effect of *X* on *Y* after accounting for mediation by *M* is denoted $c^{\prime },$ which in our case is analogously diet-specific and denoted here as $c_{D}^{\prime }$ with average direct effect $c^{\prime }=\bar{c_{d}^{\prime }}.$ (Not explicitly calculated here but used elsewhere is *c*, which would be the effect of *X* on *Y* if mediation were unmodeled.) When ab and $c^{\prime }$ have opposite signs, mediation by way of *M* acts to suppress the overall parent-of-origin effect on the outcome *Y*.

#### Linked LMMs

Our mediation model for the effect of a gene-expression-mediator *z* on an outcome *y* is specified via two linked LMMs as:

$$f\left(y_{mi}\right)=\mathrm{int}{\mathrm{cov}}_{mi}+{\text{diet}}_{d\left[m\right]}+{\text{POE}}_{s\left[m\right]}+\text{diet}.\text{by}.{\text{POE}}_{\left(sd\right)\left[m\right]}+\underset{\text{effect of }z\text{ on }y}{\underset{︸}{b\cdot f^{\left(z\right)}\left(z_{mi}\right)}}+{\text{dam}}_{m}+\varepsilon_{mi},$$ (5)

where $f^{\left(z\right)}$ denotes a transformation that may be different from *f*, *b* is the effect of mediator *z* on *y*, and the combined contribution of POE and diet.by.POE provides the direct effect $c_{D}^{\prime }.$ Meanwhile, mediator *z* is simultaneously modeled as:

$$f^{\left(z\right)}\left(z_{mi}\right)={\mathrm{intcov}}_{mi}^{\left(z\right)}+{\text{diet}}_{d\left[m\right]}^{\left(z\right)}+{\text{POE}}_{s\left[m\right]}^{\left(z\right)}+\text{diet}.\text{by}.{\text{POE}}_{\left(sd\right)\left[m\right]}^{\left(z\right)}+{\text{dam}}_{m}^{\left(z\right)}+\varepsilon_{mi}^{\left(z\right)},$$ (6)

where, for example, the notation ${\mathrm{intcov}}_{mi}^{\left(z\right)}$ means the same regression input as ${\mathrm{int}}_{mi}$ but with regression coefficients specific to mediator *z* rather than outcome *y*, and the combined contribution of ${\text{POE}}^{\left(z\right)}$ and $\text{diet}.\text{by}.{\text{POE}}^{\left(z\right)}$ provides the effect $a_{d}.$ Specifically, the correspondence of Eq 5 and Eq 6 to the more general mediation analysis is as follows:

$$X_{mi}=I\left(s\left[m\right]=\text{NOD}\right)\left(\text{i}.\text{e., 1 if NOD maternalstrain,0 }\text{o}/\text{w}\right)$$

$$M_{mi}=f^{\left(z\right)}\left(z_{mi}\right)$$

$$Y_{mi}=f\left(y_{mi}\right)$$

b=b     (effect of zony)

$$a_{d}={\text{POE}}_{\text{NOD}}^{\left(z\right)}+\text{diet}.\text{by}.{\text{POE}}_{\text{NOD},d}^{\left(z\right)}$$

$$c_{d}^{\prime }={\text{POE}}_{\text{NOD}}+\text{diet}.\text{by}.{\text{POE}}_{\text{NOD},d}$$

$$a=\bar{a_{d}}\;\;\;\;\left(\text{all-diets-average POE on }z\right)$$

$$ab=\bar{a_{d}}b\;\;\;\;\left(\text{all-diets-average mediated POE on }y\right)$$

$$c^{\prime }=\bar{c_{{}_{d}}^{\prime }}\;\;\;\;\left(\text{all-diets-averag edirect POE on }y\right)$$

where ${\text{POE}}_{\text{NOD}}$ is the effect of switching from an NOD mother to a B6 mother, and $\text{diet}.\text{by}.{\text{POE}}_{\text{NOD},\;d}$ is the additional effect of this for diet *d*.

#### Transformations, expression adjustment, priors, and MCMC sampling.

Prior to fitting the Bayesian mediation model, all candidate mediators and outcomes were transformed using the same process as described earlier; *i.e.*, transforms were chosen to ensure normality using the frequentist, mediation-free models (Appendix B, C). Additionally, akin to the mediation-free microarray analysis, surrogate variable effects (Appendix D) were regressed out of every gene’s expression prior to mediation modeling. However, unlike the mediation-free analysis of expression, breeding batch and pipeline were *not* regressed out, and were included as nuisance effects on mediator and outcome in the mediation model. Priors were specified as follows, noting that *M* and *Y* by construction have means of 0 and standard deviation 1: fixed effects (*i.e.*, all effects except dam) were given priors of $\text{N}\left(0,5^{2}\right);$ and the random effect of dam was modeled as drawn from $\text{N}\left(0,\tau^{2}\right)$ with $\tau^{2}∼\text{Unif}\left(0,25\right).$ Model fitting proceded by running a single MCMC chain for 16,000 timesteps, of which the first 3,200 were discarded (*i.e.*, as burn-in), and the last 12,800 were retained for estimation.

#### Combined Tail Probability: a statistic to quantify aggregate mediation

To quantify the extent to which a given gene’s expression mediated POE on multiple outcomes, we use a statistic inspired by the Fisher combined p-value that we refer to as the “Combined Tail Probability” (CTP). The CTP is the probability that a value drawn from $\chi_{2K}^{2}$ is at least as extreme as the statistic $T=-2\sum_{k}^{K}\log\left(p_{k}\right),$ where *K* is the number of outcomes tested for mediation, and $p_{k}$ is the CTP for the mediator’s indirect effect on outcome *k*. Although the implicit distributional assumption is not strictly justified, the CTP associated with *T* provides a statistic for evaluating which mediators are strongest in aggregate.
